# Dopamine receptor D3 signalling in astrocytes promotes neuroinflammation

**DOI:** 10.1186/s12974-019-1652-8

**Published:** 2019-12-06

**Authors:** Andro Montoya, Daniela Elgueta, Javier Campos, Ornella Chovar, Paulina Falcón, Soledad Matus, Iván Alfaro, María Rosa Bono, Rodrigo Pacheco

**Affiliations:** 1 Fundación Ciencia & Vida, Avenida Zañartu #1482, Ñuñoa, 7780272 Santiago, Chile; 2 Facultad de Medicina y Ciencia, Universidad San Sebastián, Providencia, 7510157 Santiago, Chile; 3 Center for Geroscience, Brain Health and Metabolism, 7800003 Santiago, Chile; 4 Instituto de Ciencias e Innovación en Medicina, Facultad de Medicina, Clínica Alemana Universidad del Desarrollo, Las Condes, 7590943 Santiago, Chile; 5 Departamento de Biología, Facultad de Ciencias, Universidad de Chile, 7800003 Santiago, Chile; 6 Departamento de Ciencias Biológicas, Facultad de Ciencias de la Vida, Universidad Andres Bello, 8370146 Santiago, Chile

**Keywords:** Neuroinflammation, Astrocytes, Microglia, Dopamine receptors

## Abstract

**Background:**

Neuroinflammation constitutes a pathogenic process leading to neurodegeneration in several disorders, including Alzheimer’s disease, Parkinson’s disease (PD) and sepsis. Despite microglial cells being the central players in neuroinflammation, astrocytes play a key regulatory role in this process. Our previous results indicated that pharmacologic-antagonism or genetic deficiency of dopamine receptor D3 (DRD3) attenuated neuroinflammation and neurodegeneration in two mouse models of PD. Here, we studied how DRD3-signalling affects the dynamic of activation of microglia and astrocyte in the context of systemic inflammation.

**Methods:**

Neuroinflammation was induced by intraperitoneal administration of LPS. The effect of genetic DRD3-deficiency or pharmacologic DRD3-antagonism in the functional phenotype of astrocytes and microglia was determined by immunohistochemistry and flow cytometry at different time-points.

**Results:**

Our results show that DRD3 was expressed in astrocytes, but not in microglial cells. DRD3 deficiency resulted in unresponsiveness of astrocytes and in attenuated microglial activation upon systemic inflammation. Furthermore, similar alterations in the functional phenotypes of glial cells were observed by DRD3 antagonism and genetic deficiency of DRD3 upon LPS challenge. Mechanistic analyses show that DRD3 deficiency resulted in exacerbated expression of the anti-inflammatory protein Fizz1 in glial cells both in vitro and in vivo.

**Conclusions:**

These results suggest that DRD3 signalling regulates the dynamic of the acquisition of pro-inflammatory and anti-inflammatory features by astrocytes and microglia, finally favouring microglial activation and promoting neuroinflammation.

## Background

Emerging evidence indicates that neuroinflammation plays a pivotal role in the cascade of events leading to neuronal death and progression of neurodegenerative disorders such as Alzheimer’s disease and Parkinson’s disease [1]. Neuroinflammation and consequent neurodegeneration can be also triggered by systemic inflammation, such as the case of sepsis [2, 3]. Since microglia might stimulate innate as well as adaptive immunity in the central nervous system (CNS), these cells play a major role in neuroinflammation [4]. Consequently, the functional phenotype acquired by microglia determines whether surrounding neurons survive or die. Accordingly, depending on the integration of different molecular cues, microglial cells might acquire two different phenotypes, including an inflammatory M1 phenotype and an anti-inflammatory M2 phenotype [5]. M1 microglia generates a neurotoxic microenvironment by producing glutamate, TNF-α and reactive oxygen and nitrogen species [6–8], whilst M2 microglia produces anti-inflammatory mediators and neurotrophic factors, such as insulin-like growth factor-1 (IGF-1), brain-derived neurotrophic factor (BDNF), glial-derived neurotrophic factor (GDNF) and found in inflammatory zone 1 (Fizz1) thus inducing a supportive microenvironment for neurons [1, 9–13].

Several studies have shown that acquisition of different functional phenotypes of microglia can be strongly influenced by soluble mediators such as cytokines, neurotransmitters and by the action of astrocytes [4]. Accordingly, astrogliosis constitutes a neuropathological feature in Parkinson’s disease [14], Alzheimer’s disease [15] as well as in sepsis-associated encephalopathy [16]. This data supports the notion that astrocytes might be active players in neurodegeneration associated to these neurodegenerative disorders. Interestingly, analyses of necropsies of Parkinson’s disease patients have shown that neuronal death inversely correlates with the number of activated astrocytes [17], thereby supporting the notion that astroglial activation in Parkinson’s disease might play a beneficial role ameliorating neurodegeneration.

During the last decade, dopamine has been shown to be a major regulator of inflammation. Accordingly, dopamine receptors (DRs, DRD1-DRD5) have been found expressed in the immune system where they exert a complex regulation of immunity [18]. An increasing number of studies analysing human cells in vitro or using in vivo approaches in animal models have revealed a complex regulation of immunity exerted by dopamine. Integrating the knowledge acquired by those studies, the evidence indicates that stimulation of low-affinity DRs, for instance DRD1 and DRD2, are coupled to anti-inflammatory mechanisms, thereby dampening inflammation [19, 20]. Conversely, signalling triggered by high-affinity DRs, including DRD3 and DRD5, have been found consistently to promote inflammation [21–26].

Of note, DRs have been found not only in cells of the adaptive immune system but also in cells belonging to the innate immunity, including glial cells. In this regard, it has been shown that human microglia express DRD1-DRD4 [27], whilst DRD1, DRD2, DRD4 and DRD5 have been found in rat microglia [28]. On the other hand, all five DRs have been detected in rat astrocytes obtained from basal ganglia [29]. In the case of mouse, some discrepancies have been found in the expression of DRs by glial cells depending on the mouse strain. In this regard, all DRs have been found expressed in astrocytes and microglial cells in the outbreed NMRI strain [30]. Conversely, only DRD1 and DRD5, but not DRD3 have been detected in microglial cells obtained from the inbreed C57BL/6 strain [28, 31]. In addition, DRD2 and DRD3 have been found in astrocytes obtained from C57BL/6 mice [20, 31].

Interestingly, decreased dopamine levels have been associated with inflammatory processes, such as those found in the substantia nigra of Parkinson’s disease patients or the gut mucosa of inflammatory bowel disease patients [32]. This evidence strongly suggests that signalling triggered by high-affinity DRs becomes relevant in inflammation. According to this notion, we and other authors have recently shown that genetic deficiency of DRD3, which displays the highest affinity for dopamine, attenuates neuroinflammation and the consequent neurodegeneration on a murine model of Parkinson’s disease induced by acute intoxication with 1-methyl-4-phenyl-1,2,3,6-tetrahydropyridine (MPTP) [33, 34]. In the same direction, using a pharmacologic approach, we have found that systemic antagonism of DRD3 attenuates microglial activation and neuronal loss of the nigrostriatal pathway in two different animal models of PD, including those models of parkinsonism induced by chronic administration of MPTP or by the stereotaxic injection of 6-hydroxydopamine [25, 31]. Thus, this data indicates a prominent role of DRD3 in the control of neuroinflammation and the consequent neurodegeneration.

In this study, we aimed to evaluate how DRD3 signalling affects the dynamic of glial activation and the underlying mechanism. To this end, we used genetic and pharmacologic approaches to inhibit DRD3 signalling in a mouse model of neuroinflammation induced by systemic LPS treatment. Moreover, for mechanistic analyses, we used an in vitro approach to study the role of DRD3 signalling confined to glial cells in response to inflammatory or anti-inflammatory stimuli. Our results suggest that DRD3 signalling in astrocytes regulates the dynamic of the acquisition of pro-inflammatory and anti-inflammatory features by astrocytes and microglia, finally favouring the development of neuroinflammation.

## Materials and methods

### Animals

Wild-type (WT) C57BL/6 adult male mice (25–30 g) of 3 months of age were obtained from Jackson Laboratories (Bar Harbor, ME, USA). Adult male DRD3-knockout (DRD3KO) mice (25–30 g) of 3 months of age in the C57BL/6 background were kindly donated by Dr. Marc Caron [35]. For experiments using glial cell cultures, C57BL/6 mouse pups were used 1–4 days after birth. Four to five mice per cage were housed at 21 °C in a humidity-controlled environment, on a 12/12 h light/dark cycle with lights on at 8 AM, with ad libitum access to food and water. All mice were maintained and manipulated according to institutional guidelines at the pathogen-free facility of the Fundación Ciencia & Vida. The experimental design was approved by the Ethical Committee for Animal Testing of the Fundación Ciencia & Vida.

### In vivo treatments with LPS and PG01037

To induce systemic inflammation and a consequent neuroinflammation, 5 mg/kg of LPS (Sigma-Aldrich, St. Louis, MO) were i.p. administered as described before [36]. Three, 4 or 24 h after LPS injections, mice were sacrificed and the midbrain fraction was extracted as detailed below. In some experiments, mice received i.p. injections of PG01037 (30 mg/kg; Tocris) dissolved in PBS 1 h before LPS administration, a dose that has been proven to exert a therapeutic effect attenuating neurodegeneration in mouse models of Parkinson’s disease [31].

### Tissue processing

For flow cytometry analysis or RNA extraction animals were sacrificed with an overdose of 5% isoflurane (Sigma-Aldrich) and transcardially perfused with PBS. Brains were rapidly removed, submerged in cold Hank’s balanced salt solution (HBSS) while different structures were dissected, and immediately processed for subsequent analysis. For immunohistochemical analysis, mice were transcardially perfused with 0.9% NaCl, followed by 4% paraformaldehyde (Sigma-Aldrich).

### Immunohistochemical and immunofluorescence analyses of tissue preparations

To carry out immunohistochemistry analysis, free floating sections (40 μm thick) were processed at the same time in each experiment. Sections were washed with PBS and endogenous peroxidase activity was inactivated by incubation with 0.03% H_2_O_2_ in methanol (Sigma-Aldrich) for 30 min. After washing three times with PBS, the tissue was incubated for 40 min with blocking solution [4% goat serum, 0.05% Triton X-100 (Sigma-Aldrich) and 4% BSA (Merck, Darmstadt, Germany) in PBS], and exposed overnight to the primary antibodies diluted in blocking solution at room temperature. The primary antibodies used were rabbit anti-GFAP antibody (1:500; Abcam [EPR1034Y], Cambridge, UK) and rabbit anti-Iba1 antibody (1:1000; Abcam [EPR16588], Cambridge, UK). After washing, sections were incubated with biotinylated goat anti-rabbit antibody (1:500; Jackson ImmunoResearch Laboratories, West Gore, PA, USA) in blocking solution for 2 h at room temperature. Sections were washed and then incubated with peroxidase-conjugated avidin (1:5000; Sigma-Aldrich) for 90 min at room temperature followed by incubation with 0.05% diaminobenzidine (Sigma-Aldrich) in 0.03% H_2_O_2_/Trizma-HCl buffer (pH 7.6). To evaluate the extent of astrogliosis, the mean of glial fibrillary acidic protein (GFAP)-associated immunoreactivity was analysed in areas of interest (660 μm × 877 μm) in five striatum sections per mouse and quantified as the integrated density using the ImageJ software. To determine the extent of activated microglia, the mean number of Iba-1^high^ reactive microglia displaying ameboid shape was quantified in areas of interest of 660 μm × 877 μm in five striatum sections per mouse.

To perform immunofluorescence analysis, free floating sections (40 mm thick) were processed at the same time in each experiment. Tissue sections were first incubated with blocking solution [10% normal goat serum (Jackson ImmunoResearch Laboratories, West Gore, PA, USA), 0.3% Triton X-100 (Sigma-Aldrich) and 5% BSA (Merck, Darmstadt, Germany) in PBS] for 1 h and then exposed overnight to the primary anti-DRD3 antibody diluted in blocking solution (1:100) at room temperature. According to the manufacturer’s recommendations, the rabbit anti-DRD3 antibody (ADR-003, Alomone labs) was directly used or pre-incubated with the antigenic peptide DRD3_15-29_ (CGAENSTGVNRARPH) used to develop the antibody (in a mixture of 0.8 mg/ml of anti-DRD3 antibody and 0.4 mg/ml of peptide) for 30 min as a control to abolish the specific immunostaining. After washing three times with PBS, tissue sections were incubated 2 h at room temperature with the secondary antibody goat anti-rabbit AlexaFluor488 antibody (1:200). Afterward, samples were washed three times with PBS and incubated in blocking solution at room temperature for 1 h. Tissue sections were washed with PBS and incubated with rabbit anti-GFAP-PE antibody (1:100; clone 1B4, BD Pharmigen) for 1 h. After washing three times with PBS, tissue sections were mounted using DAKO Fluorescent Mounting Medium (Dako, California, USA). The nuclei of the cells were marked with DAPI. Images were acquired using a fluorescence microscope Olympus FLUOVIEW FV1200 (Olympus, Tokio, Japan).

### Microscopy imaging and image analysis of tissue immunofluorescence

Image stacks of brain slices immunostained for DRD3, GFAP and Nuclei were acquired in a FV1200 Olympus confocal microscopy using a × 40 AN1.3 oil immersion objective at 2048 × 2048 pixel resolution with a Z step of 10 × 0.5 μm using corresponding excitation and emission settings for alexa488 (DRD3), PE (GFAP) and DAPI (Nuclei). To analyse the extent of DRD3 immunostaining in areas were GFAP^+^ cells were present, maximal intensity Z projections of images were obtained and intensity correlation analysis was carried out using the JaCoP Plugin of the ImageJ software [37].

### Glial cultures

Mixed glial cultures were generated from mouse pups 1–4 days after birth. Brains were removed from skull, meninges were removed and the striatum and midbrain regions were extracted. The tissue was dissociated using trypsin 0.01% in HBSS during 10 min. Afterwards, cells were washed, resuspended in DMEM/F12 (HyClone, Utah, USA) media supplemented with 10% foetal bovine serum (FBS; Gibco, New York, USA) and tissue was completely dissociated by mechanical disaggregation using a glass Pasteur pipettes. Cells obtained from four brains were seeded onto T-75 flask in 10 ml of DMEM/F12 supplemented with 2.5 mM l-glutamine and 10% FBS. After incubation for 24 h, the medium and tissue were removed and replaced by fresh medium. Afterwards, half of the volume of media was renovated every 72 h until cells reached 90% confluence. At this point, cells were plated in a new T-75 flask. The mixed glial culture was maintained at 37 °C in humidified atmosphere of 5% CO_2_ and used for experiments after 14–28 days. Cells were left untreated or treated with 1 μg/ml LPS or with 25 ng/ml IL-4 for 24 h and then RNA was extracted as indicated below.

Microglia were obtained from 14 to 28 days mixed glial culture by mechanical extraction using a horizontal rotating shaker at 200 rpm for 2 h. Microglial cells contained in the supernatant were centrifuged at 500 g, resuspended in DME/F12 media (supplemented with 2.5 mM l-glutamine and 10% FBS) and plated in T-25 flask. The purity of the microglial cells obtained was evaluated by CD11b immunostaining (≥ 98% CD11b^+^ cells).

Purification of astrocytes was carried out from the mixed glial culture by depletion of microglial cells. For this purpose, after 7 days of incubation, mixed glial cultures were shaked at 180 rpm overnight twice a week throughout 2 weeks and the supernatant containing microglial cells was discarded and replaced by fresh media every time. Afterwards, astrocytes layer was detached by incubation with trypsin 0.01% in HBSS for 30 min, and seeded onto T-75 flask with DME/F12 media supplemented with 10% FBS. The purity of astrocytes culture was evaluated by GFAP immunostaining (≥ 96% GFAP^+^ cells).

### Quantitative RT-PCR

Total RNA extracted from cells using the Total RNA EZNA kit (Omega Bio-Tek, Norcross, GA, USA) was digested with DNase using the TURBO DNA-free kit (Ambion, Thermo Fisher Scientific) and 1 μg of RNA was used to synthesize cDNA utilizing M-MLV reverse transcriptase, according to manufacturer’s instructions (Life Technologies). Quantitative gene expression analysis was performed using Brilliant II SYBR Green QPCR Master Mix (Agilent Technologies, Santa Clara, CA, USA), according to the manufacturer’s recommendations. Primers were used at a concentration of 0.5 μM. We used 40 PCR cycles as follows: denaturation 30 s at 95 °C, annealing 30 s at 60 °C and extension 60 s at 72 °C. Expression of target genes was normalized to *Gapdh.* The sequences of the primers used are indicated in Table 1.

**Table 1:** Sequence of primers used for quantitative RT-PCR analyses of inflammatory and anti-inflammatory markers

Gene	Forward primers 5′ → 3′	Reverse primers 5′ → 3′
*arg1*	TGACATCAACACTCCCCTGACAAC	GCCTTTTCTTCCTTCCCAGCAG
*fizz1*	ACCTTTCCTGAGATTCTGCCCC	CAGTGGTCCAGTCAACGAGTAAGC
*ym1*	GGCTACACTGGAGAAAATAGTCCCC	CCAACCCACTCATTACCCTGATAG
*nrf2*	TCACACGAGATGAGCTTAGGGCAA	TACAGTTCTGGGCGGCGACTTTAT
*nos2*	GGTCTTTGACGCTCGGAACTGTAG	CACAACTGGGTGAACTCCAAGGTG
*igf-1*	GGACCAGAGACCCTTTGCGGGG	GGCTGCTTTTGTAGGCTTCAGTGG
*nt-3*	CCGGTGGTAGCCAATAGAACC	GCTGAGGACTTGTCGGTCAC
*ngf*	TGATCGGCGTACAGGCAGA	GAGGGCTGTGTCAAGGGAAT
*bdnf*	CCTTACTATGGTTATTTCATACTTCGGTT	TCAGCCAGTGATGTCGTCGTC
*gdnf*	GCCACCATTAAAAGACTGAAAAGG	GCCTGCCGATTCCTCTCTCT
*drd3*	GAACTCCTTAAGCCCACCAT	GAAGGCCCCGAGCACAAT
*gadph*	TCCGTGTTCCTACCCCCAATG	GAGTGGGAGTTGCTGTTGAAG

### Flow cytometry and immunofluorescence analysis of glial cells

Brain sections containing midbrain and striatum from adult mice were minced and then digested by Colagenase Type IV 1 mg/ml (Gibco, New York, USA) and DNase I 0.25 mg/ml (Roche, Mammheim, Germany). After tissue digestion, cells were filtered through 70-μm pore cell-strainer to obtain a single-cell suspension. For intracellular immunolabeling of phenotypic markers in astrocytes, cells were first fixed and permeabilized. Then, astrocytic markers were immunostained using the following fluorochrome-coupled antibodies: PE-coupled anti-mouse GFAP mAb (BD, California, USA), FITC-coupled anti-mouse inducible nitric oxide synthase (iNOS) mAb (BD) and APC-coupled anti-mouse arginase-1 mAb (R&D Systems Inc., Minneapolis, USA). Detection of microglial markers was performed with APC-Cy7 coupled anti-mouse CD16/32 mAb, APC-coupled anti-mouse CD206 mAb, PE-Cy7-coupled anti-mouse CD11b mAb and PE-coupled anti-mouse CD45 mAb, all of them obtained from Biolegend (San Diego, CA, USA). All analyses were performed in living cells using the Zombie Aqua (ZAq) fixable viability kit (Biolegend) in the ZAq^-^ population. Flow cytometry analysis was performed with identical instrument settings on a FACS Canto II (BD). Data were analysed using the FlowJo software (Tree Star).

For immunofluorescence analysis, microglia or astrocytes cultures were mounted onto cover glasses and fixed with 4% paraformaldehyde, permeabilised using 0.1% Triton X-100 in PBS and then saturated with blocking solution containing 5% BSA and 0.1% Triton X-100 in PBS. Afterward, cells were incubated over night at 4 °C in incubating solution (1% BSA and 0.1% Triton X-100 in PBS) containing the following primary antibodies: rat anti-mouse CD11b (1:500; Serotec, Raleigh, USA), rabbit anti-mouse GFAP (1:1000; Synaptic System, Goettingen, Germany) and rabbit anti-mouse DRD3 (1:100; Abcam, Boston, USA). After three washes with incubating solution, the cells were incubated 1 h at room temperature with secondary antibodies goat anti-rabbit Alexa-fluor 488 (for DRD3 immunostaining) or goat anti-rabbit Alexa-fluor 546 (for GFAP immunostaining) (1:200; Invitrogen, Oregon, USA) and goat anti-rat DyLigth 488 (for CD11b immunostaining) (1:200; Jackson ImmunoResearch Laboratories), and after several washes, the cells were mounted using DAKO fluorescent mounting medium (Dako, California, USA). The nuclei of the cells were marked with DAPI. Images were acquired with an inverted fluorescence microscope Olympus IX71 (Olympus, Tokyo, Japan) coupled to a power supply unit (Olympus U-RFL-T).

### Determination of Fizz1, TNFα and IL-1β production by ELISA

Cells cultures were incubated in the presence of indicated stimuli for 24 h and the cytokine production was evaluated in the supernatant. For this purpose, serial dilutions were made from the original supernatants and the concentration of Fizz1, IL-1β and TNFα were quantified using commercial kits purchased from PeproTech (for Fizz1) and Invitrogen (for IL-1β and TNFα) following the manufacturer’s recommendations.

### Statistical analysis

All values are expressed as the mean ± SEM. Statistical analysis were performed with two-tailed unpaired Student’s *t* test when comparing only two groups and with one-way ANOVA followed by Tukey’s post-hoc test when comparing more than two groups with only one variable (treatment or genotype). To analyse differences in experiments comparing different genotypes and different treatments, two-way ANOVA followed by Sidak’s post-hoc test was performed. All analyses were carried out using the GraphPad Prism 6 Software. *P* values < 0.05 were considered significant.

## Results

### DRD3 is selectively expressed in astrocytes but not in microglial cells

**Fig. 1:**
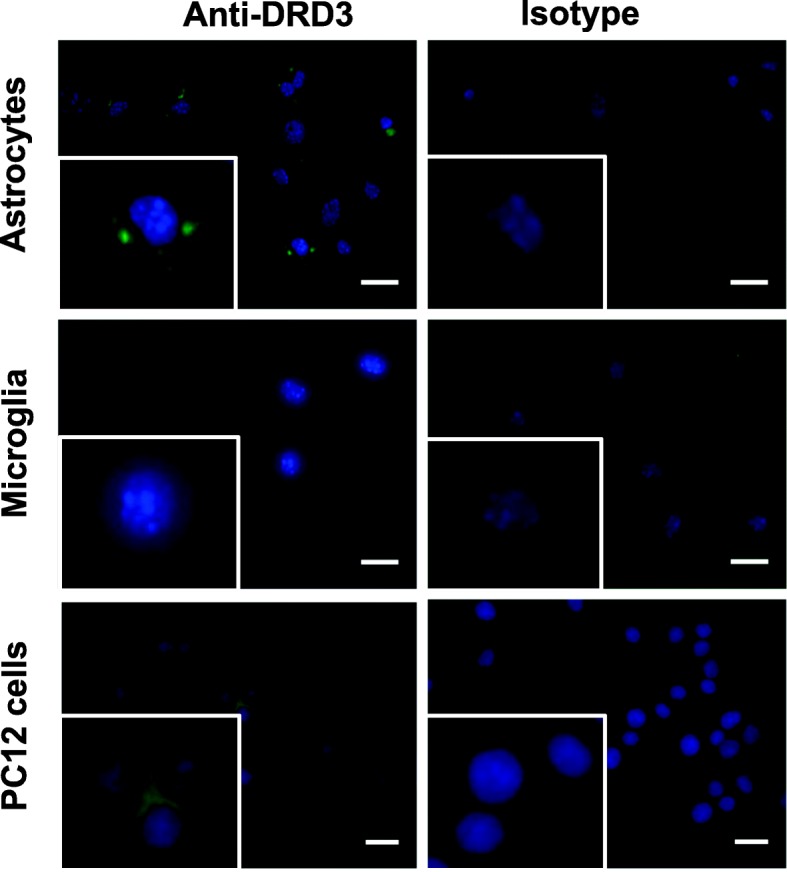
DRD3 is selectively expressed in astrocytes but not in microglial cells. Astrocyte (top panels) and microglial (middle panels) cultures were obtained from wild-type mice as described in materials and methods. PC12 cell line was used as a positive control for DRD3 expression (bottom panels). Cells were immunostained with anti-DRD3 antibody (left panels) or an isotype-matched control (right panels) primary antibody followed by an Alexa488-coupled secondary antibody and the immunofluorescence (green) associated was analysed by fluorescence microscopy. Nuclei were stained with DAPI (blue). Representative photomicrographs are shown. A higher magnification section is inserted in the bottom left corner of each image. Bar, 10 μm

We previously appreciated a selective expression of the *drd3* transcript in primary astrocytes, which was not detected in primary microglial cells [31]. To evaluate whether this differential expression is actually observed at the protein level, we performed immunofluorescence analyses in which we compared the expression of DRD3 in primary cultures of microglia and astrocytes obtained from wild-type (WT) C57BL/6 mice. In these analyses, the PC12 cell line, which has been shown to express DRD3 [38], was used as a positive control. The results show significant immunoreactivity associated to the anti-DRD3 antibody in comparison to the isotype matched control in both primary astrocytes and PC12 cells, but not in primary microglial cells (Fig. 1). Thus, these results suggest that, in agreement with our previous analyses at the level of *drd3* transcript, DRD3 is selectively expressed in astrocytes but not in microglial cells.

**Fig. 2:**
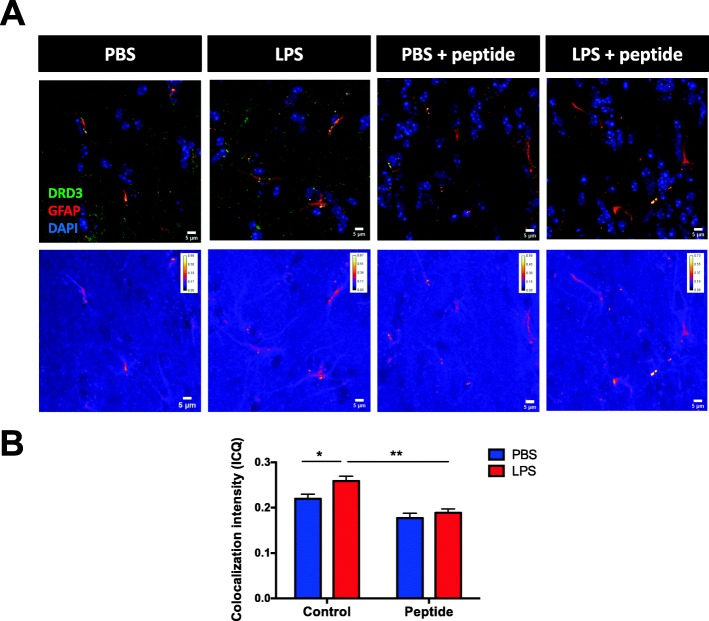
DRD3 specific immunoreactivity is associated to astrocytes in the mouse brain upon systemic inflammation. Wild-type mice received an i.p. injection of PBS or LPS (5 mg/kg) and 3 h later were sacrificed and immunofluorescence analysis was performed in brain slices. To determine the specific DRD3 immunostaining, a polyclonal anti-DRD3 antibody was pre-incubated or not with the peptide DRD3_15-29_ (CGAENSTGVNRARPH) used as immunogen to develop the antibody. Afterward, brain sections were incubated with untreated anti-DRD3 or with anti-DRD3 pre-incubated with peptide DRD3_15-29_, followed by incubation with the AlexaFluor488-coupled secondary antibody (green). Samples were subsequently immunostained with anti-GFAP-PE (red) antibody and nuclei were stained with DAPI (blue). **a** Representative images of the subventricular zone are shown (top panels; bar, 5 μm). Pseudocolored images showing areas in which DRD3-associated intensity correlates positively with GFAP-associated intensity are shown in bottom panels (bar, 5 μm). **b** Intensity correlation analysis of confocal microscopy images obtained from DRD3 and GFAP immunostaining. Colocalization intensity was determined as the intensity correlation quotients (ICQ). Data from four mice per group is shown. Values are the mean ± SEM. *p < 0.05; **p < 0.01; by two-way ANOVA followed by Sidak’s post-hoc test

To evaluate whether DRD3 immunoreactivity is also associated to astrocytes in vivo upon inflammatory conditions, WT mice were treated with systemic LPS (5 mg/kg) or vehicle (PBS) and 3 h later sacrificed and the immunoreactivity for DRD3 and GFAP were analysed in brain slices, since previous results have shown that neuroinflammation is already evident in these conditions [36]. For this purpose, we first analysed GFAP expression in different areas of the brain in mice treated with LPS (Additional file 1: Figure S1). Despite high GFAP expression was observed in different areas of the brain (Additional file 1: Figure S1), subventricular zone was chosen to further analysis of the co-expression of GFAP and DRD3, as individual GFAP^+^ cells were clearly identified in this region. To confirm the specificity of the immunoreactivity associated to the anti-DRD3 antibody used in this analysis, this antibody was pre-incubated with the immunogen used to develop the antibody, the peptide DRD3_15-29_. Of note, we have recently demonstrated how the immunoreactivity associated to this anti-DRD3 antibody is lost in DRD3-deficient cells [25]. The results show that DRD3-immunoreactivity is in part associated to GFAP-immunoreactivity (Fig. 2a, top panels). Furthermore, when the specific immunoreactivity was abrogated by pre-incubating the anti-DRD3 antibody with the DRD3_15-29_ peptide, most DRD3-immunoreactivity disappeared (Fig. 2a, top panels). In addition, to quantify the degree of colocalization, we used the intensity correlation analysis method [37]. As observed in pseudocolored images (Fig. 2a, bottom panels), DRD3 intensity variations synchronize better with stronger GFAP intensities (GFAP^+^ cells) than with background random intensities in surrounding structures. Interestingly, the quantification of intensity correlation quotients (ICQ) shows a significant increase in the colocalization of DRD3 and GFAP when mice were treated with systemic LPS in comparison with those mice treated with PBS (Fig. 2b). Moreover, the degree of colocalization of GFAP and DRD3 immunostaining in brain samples obtained from LPS-treated mice was significantly lower when the anti-DRD3 antibody was pre-incubated with the peptide DRD3_15-29_ (Fig. 2b), indicating that GFAP^+^ cells colocalize with the specific DRD3 immunoreactivity in LPS-treated mice. Of note, the degree of colocalization of DRD3 and GFAP immunoreactivity was not significantly different in the presence or in the absence of the peptide DRD3_15-29_ in samples obtained from PBS-treated mice (Fig. 2b), suggesting that there is no significant DRD3 colocalizing with GFAP^+^ cells in steady-state. Together, these results suggest that, at least in part, specific DRD3 immunoreactivity is associated with astrocytes in the mouse brain upon systemic inflammation.

### The inhibition of DRD3 signalling attenuates microglial activation in the brain of mice undergoing systemic inflammation induced by LPS

**Fig. 3:**
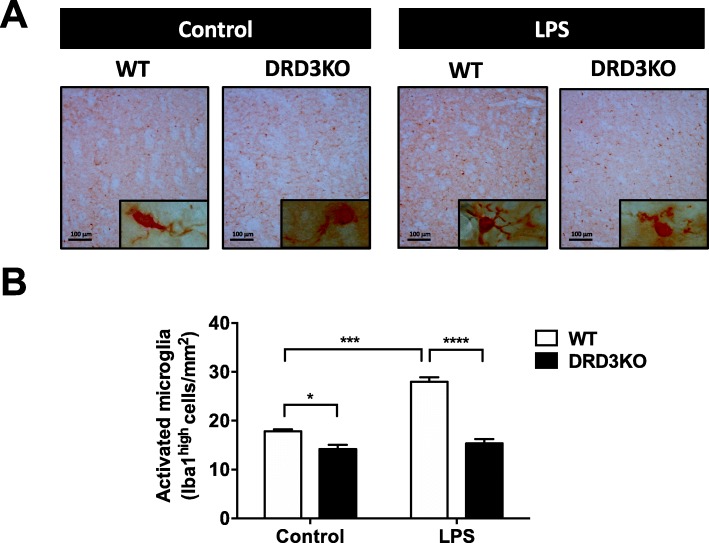
DRD3 deficiency results in attenuated microglial activation upon systemic LPS treatment. Wild-type (WT) or DRD3 knockout (DRD3KO) mice received an i.p. injection of LPS (5 mg/kg) or PBS. After 3 h, mice were sacrificed, and microglial activation was analysed by immunohistochemical analysis of Iba1 in the striatum. **a** Representative overview images at low magnification (× 20) are shown. High magnification (× 100) images are inserted in the bottom-right corner of each overview image. **b** Quantification of the density of Iba1^high^ cells with ameboid shape. Data from five to six mice per group is shown. Values are the mean ± SEM. *p < 0.05; ***p < 0.001; ****p < 0.0001 by two-way ANOVA followed by Sidak’s post-hoc test

**Fig. 4:**
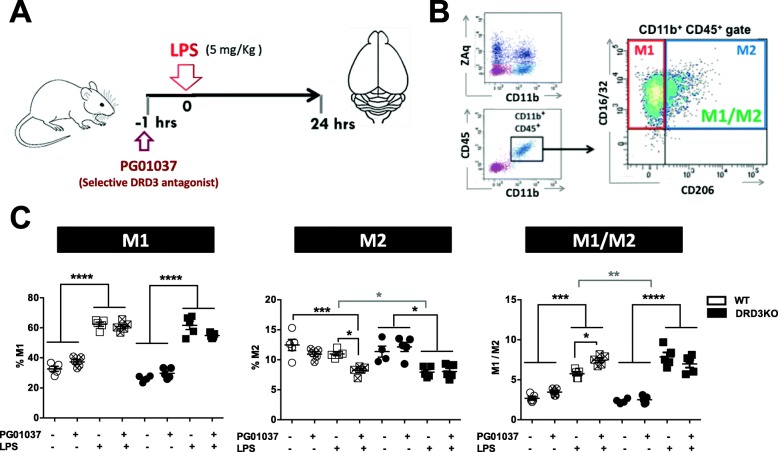
Genetic deficiency or pharmacologic antagonism of DRD3-signalling attenuates the acquisition of M2-phenotype by microglial cells in the midbrain of mice undergoing systemic inflammation induced by LPS. Wild-type (WT) or DRD3 knockout (DRD3KO) mice were pre-treated or not with an i.p. injection of a DRD3-selective antagonist (PG01037; 30 mg/kg) and 1 h later received an i.p. injection of LPS (5 mg/kg) or PBS. Twenty-four hours after LPS administration, the midbrain/striatum structures were isolated, disaggregated, and M1 and M2 phenotypes were analysed in microglial cells by flow cytometry. **a** Schematic illustration of the experimental design. **b** Gating strategy used to analyse the M1 (CD16/32^+^CD206^-^ cells) and M2 (CD16/32^+^CD206^+^ cells) phenotypes in living (ZAq^−^) microglial cells (CD11b^+^ CD45^+^). **c** Quantification of the frequencies of M1 (left panel) and M2 (middle panel) phenotypes and the M1-to-M2 ratio (right panel). Data from five mice per group is shown. Each symbol represents a WT (white) or a DRD3KO (black) animal. In each experimental group, the line and error bars represent the mean ± SEM, respectively. *p < 0.05; **p < 0.01; ***p < 0.001; ****p < 0.0001 by one-way ANOVA followed by Tukey’s post-hoc test. Black asterisks represent significant differences between treatments, whilst grey asterisks represent significant differences between genotypes

To determine the in vivo relevance of DRD3 in glial activation, we next performed a set of experiments in which neuroinflammation was induced by the systemic administration of LPS [36] in mice harbouring the genetic deficiency of DRD3 or undergoing pharmacological inhibition of DRD3 signalling. For this purpose, a DRD3 selective antagonist PG01037 was i.p. administered at 30 mg/kg, a drug that has been previously proven to cross the blood-brain barrier and a dose that exerts a therapeutic effect attenuating neurodegeneration in two different mouse models of Parkinson’s disease [31]. Accordingly, WT or DRD3KO mice were pre-treated or not with PG01037 and then systemic inflammation was induced by a single injection of LPS (5 mg/kg). Since previous studies have shown that systemic LPS administration triggers the loss of dopaminergic neurons of the nigrostriatal pathway [36, 39], we focused the analysis of neuroinflammation in the midbrain of mice treated with LPS. To validate this animal model of neuroinflammation in our hands, we first determined whether inflammatory cytokines were elevated in the brain of LPS-treated animals. Accordingly, we found that transcripts for TNF-α and IL-1β were strongly increased in the midbrain and striatum of mice 24 h after systemic LPS administration (Additional file 1: Figure S2A and B). To determine the activation of microglia, we first performed an immunohistochemical analysis of Iba1 3 h after LPS-treatment as described before [36]. The results show that after LPS treatment, WT mice present a significant increase in the percentage of microglial cells with typical activated phenotype, including amoeboid shape and high density of Iba1 expression (Fig. 3a, b). Conversely, DRD3-deficient mice presented a significant reduction in the percentage of activated microglia in steady-state conditions, and the degree of microglial activation was not changed after LPS treatment (Fig. 3a, b). Thus, these results suggest that DRD3 deficiency results in attenuated microglial activation upon systemic LPS treatment. As a complementary approach to evaluate the role of DRD3 signalling in the acquisition of inflammatory phenotype by microglial cells, we determined the M1 and M2 phenotypes acquired by microglia 24 h after LPS treatment in WT and DRD3KO mice. To this end, we evaluated surface markers defining the M1 (CD16/32^+^ CD206^−^) and M2 (CD16/32^+^ CD206^+^) phenotypes in the gate of microglial cells (CD11b^+^ CD45^+^) by flow cytometry (Fig. 4a, b) as described before [6]. The results show that the percentage of M1 microglia was not affected by genetic deficiency or pharmacologic antagonism of DRD3-signalling (Fig. 4c and Additional file 1: Figure S3). Nevertheless, the percentage of M2 phenotype in microglial cells was significantly reduced upon DRD3 antagonism in LPS-treated WT mice (Fig. 4c). Accordingly, the percentage of M2 microglia was lower in LPS-treated DRD3KO mice in comparison to LPS-treated WT mice (Fig. 4c). As expected, DRD3-antagonism had no effect in the extent of M1 or M2 percentages in microglia of DRD3-deficient mice (Fig. 4c). According to the changes observed in M2 microglia, genetic deficiency or pharmacologic antagonism of DRD3 signalling resulted in increased M1-to-M2 ratio in microglial cells upon LPS-induced neuroinflammation (Fig. 4c). To further characterize the effect of DRD3 signalling in microglial activation upon LPS-induced neuroinflammation, we also evaluated the density of surface expression of key molecular markers associated to microglial activation, including CD11b (also known as Mac-1), CD45, CD16/32 and CD206. As expected, surface density of CD11b and CD45 was increased upon LPS treatment; however, neither DRD3 antagonism in WT mice nor DRD3 deficiency affected these parameters (Additional file 1: Figure S4). Similarly, no relevant changes in surface density of CD16/32 and CD206 were observed in microglia upon genetic-deficiency or pharmacologic antagonism of DRD3 signalling (Additional file 1: Figure S4).

To further analyse the effect of DRD3 signalling in the dynamic of microglial activation, the acquisition of M1 and M2 phenotypes by microglial cells was evaluated at earlier time-points after LPS-induced neuroinflammation. For this purpose, we determined the percentage of M1 and M2 microglia 4 h after systemic LPS administration, as it has been shown that a number of pro- and anti-inflammatory cytokines produced by microglial cells peaked at this time-point after LPS-induced neuroinflammation [40]. Moreover, consistently 4 h after systemic LPS treatment constitutes a time-point in which neuroinflammation is already appreciated in our hands (Additional file 1: Figure S2B). The results show a similar extent of increased percentage of M1 phenotype and decreased percentage of M2 microglia in WT and DRD3KO mice upon LPS treatment (Additional file 1: Figure S5A). Nevertheless, according to the effect of DRD3 signalling observed at 24 h after LPS treatment (Fig. 4c), the M1-to-M2 ratio was significantly higher in DRD3-deficient mice even after 4 h of LPS-induced neuroinflammation (Additional file 1: Figure S5A). Thus, these results together suggest that DRD3 signalling would be affecting the inflammatory behaviour of microglial cells upon LPS-induced inflammation.

### DRD3 deficiency results in unresponsiveness of astrocytes upon LPS-induced neuroinflammation

**Fig. 5:**
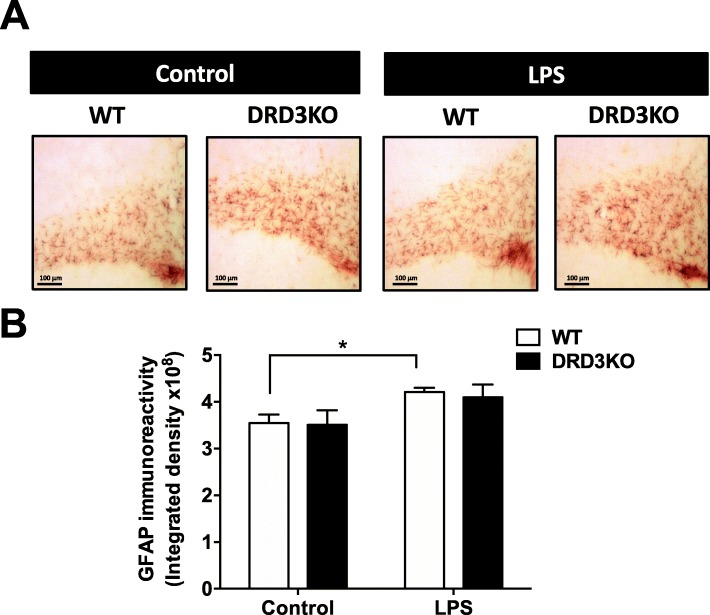
DRD3 deficiency results in unresponsiveness of astrocytes upon systemic LPS treatment. Wild-type (WT) or DRD3 knockout (DRD3KO) mice received an i.p. injection of LPS (5 mg/kg) or PBS. After 3 h, mice were sacrificed, and astrogliosis was analysed by immunohistochemical analysis of GFAP in the striatum. **a** Representative overview images at low magnification are shown (bar, 100 μm). **b** Quantification of GFAP immunoreactivity density. Data from five to six mice per group is shown. Values are the mean ± SEM. *p < 0.05 by two-way ANOVA followed by Sidak’s post-hoc test

**Fig. 6:**
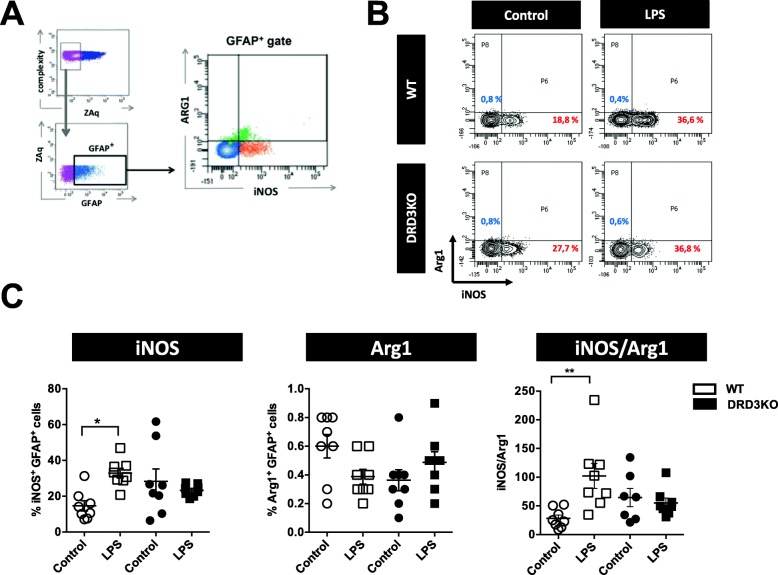
DRD3 deficiency results in an unresponsive phenotype of astrocytes in the midbrain of mice undergoing systemic inflammation induced by LPS. WT or DRD3KO mice were treated with an i.p. injection of LPS (5 mg/kg) or PBS (control). Twenty-four hours later, the midbrain/striatum structures were isolated, disaggregated and the inflammatory and anti-inflammatory phenotypes of astrocytes were analysed by flow cytometry. **a** Gating strategy used to analyse the inflammatory (iNOS^+^ cells) and anti-inflammatory (Arg1^+^ cells) phenotypes in living (ZAq^−^) astrocytes (GFAP^+^ cells). **b** Representative contour-plots indicating the percentage of pro-inflammatory glia (red numbers) and anti-inflammatory glia (blue numbers). **c** Quantification of the frequencies of inflammatory (left panel) and anti-inflammatory (middle panel) phenotypes and the inflammatory-to-anti-inflammatory ratio (right panel). Data from eight mice per group is shown. Each symbol represents a WT (white) or a DRD3KO (black) animal. In each experimental group, the line and error bars represent the mean ± SEM, respectively. *p < 0.05; **p < 0.01 by one-way ANOVA followed by Tukey’s post-hoc test

Since astrocytes might strongly regulate microglial behaviour [41] and astrocytes but not microglia express DRD3 (Fig. 1), we also aimed to evaluate whether DRD3 signalling affects the phenotype of astrocytes upon LPS-induced neuroinflammation. To determine the acquisition of activated phenotype by astrocytes, we performed immunohistochemical analyses of GFAP^+^ cells of WT and DRD3-deficient mice 3 h after LPS-treatment. The results show similar extent of astrogliosis of WT and DRD3KO mice both in control conditions or after LPS treatment (Fig. 5a, b). Despite we observed just a slight increase of GFAP immunoreactivity after LPS-treatment, only WT, but not DRD3KO mice, presented a significant increase of astrogliosis after LPS treatment (Fig. 5a, b). To gain deeper insight in the involvement of DRD3 signalling in astrocyte activation, 24 h after LPS treatment, we evaluated molecular markers defining the inflammatory (inducible nitric oxide synthase; iNOS) and anti-inflammatory (arginase 1; Arg-1) phenotypes in the gate of astrocytes (GFAP^+^) by flow cytometry (Fig. 6a) as describe before [42]. Interestingly, whereas the percentage of inflammatory phenotype of astrocytes (iNOS^+^ GFAP^+^ cells) was increased in WT mice, it was unaltered in DRD3-deficient mice after systemic LPS treatment (Fig. 6b). However, the frequency of anti-inflammatory phenotype of astrocytes was not affected in WT or DRD3KO mice at this time-point (Fig. 6b). Accordingly, the inflammatory-to-anti-inflammatory ratio of astrocytes was exclusively increased in WT mice but not in DRD3-deficient mice 24 h after LPS-induced neuroinflammation (Fig. 6b). Of note, neither inflammatory nor anti-inflammatory phenotypes were affected by DRD3 signalling at an earlier time-point (4 h) after LPS-induced neuroinflammation (Additional file 1: Figure S5B). To further characterize the effect of DRD3 signalling in astrocyte activation upon LPS-induced neuroinflammation, we also evaluated the density of expression of astrocytic molecular markers, including GFAP, iNOS and Arg-1; however, we found no differences between WT and DRD3KO mice (Additional file 1: Figure S6). Thereby, taken together, these results (Fig. 6 and Additional file 1: Figure S5B and S6) indicate that the genetic deficiency of DRD3 signalling results in an unresponsive phenotype of astrocytes upon LPS-induced neuroinflammation.

### Deficiency of DRD3 signalling results in exacerbated production of the anti-inflammatory mediator Fizz1 and decreased expression of the pro-inflammatory enzyme iNOS

**Fig. 7:**
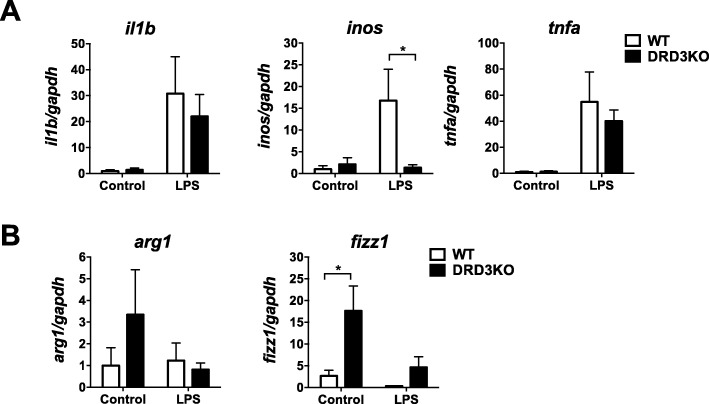
DRD3 deficiency results in altered expression of inflammatory and anti-inflammatory mediators in the midbrain of mice basally and undergoing systemic inflammation induced by LPS. WT (white) or DRD3KO (black) mice were treated with an i.p. injection of LPS (5 mg/kg) or PBS (control). Twenty-four hours later, the midbrain/striatum structures were isolated, disaggregated and the RNA was extracted and analysed by quantitative RT-PCR. Inflammatory (**a**) and anti-inflammatory mediators were evaluated (**b**). *Gapdh* transcript was used as a house keeping for normalization. Data from three to eight mice per group is shown. Values are the mean ± SEM. *p < 0.05 by two-way ANOVA followed by Sidak’s post-hoc test

To gain a deeper insight of the role of DRD3-signalling in neuroinflammation, we next aimed to evaluate a set of inflammatory and anti-inflammatory molecules in the brain of WT and DRD3-deficient mice upon systemic LPS treatment. For this purpose, we induced neuroinflammation by the i.p. administration of LPS in WT and DRD3KO mice and after 24 h the total RNA was isolated and quantitative RT-PCR analysis was performed to determine the extent of transcription of the pro-inflammatory mediators iNOS, IL-1β and TNF-α and of the anti-inflammatory molecules Arg-1 and Fizz1. The results show that DRD3 deficiency resulted in a selective and strong decrease of iNOS transcription upon LPS treatment (Fig. 7a). Furthermore, we noticed an increased transcription of Fizz1 in DRD3KO mice in basal conditions (Fig. 7b). Thus, these results indicate that despite DRD3 deficiency results in an exacerbated M1-to-M2 ratio in microglia (Figs. 4 and Additional file 1: S5A) and unresponsive phenotype in astrocytes (Figs. 5 and 6), the overall effect of the lack of DRD3 in LPS-induced neuroinflammation is an increased production of an anti-inflammatory mediator and the reduction in the extent of expression of a pro-inflammatory enzyme.

### Deficiency of DRD3 signalling in glial cells leads to enhanced expression of pro-inflammatory cytokines and exacerbated production of the anti-inflammatory mediator Fizz1 in response to inflammatory and anti-inflammatory stimuli respectively

**Fig. 8:**
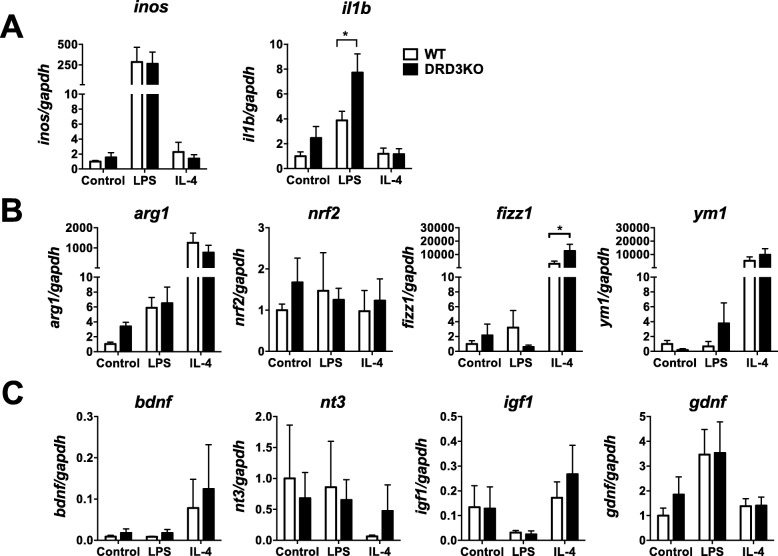
DRD3 deficiency results in a selective and strong exacerbation in the production of fizz1 in mixed glial culture in response to IL-4. Mixed glial cultures were generated from the midbrain/striatum structures obtained from WT (white) or DRD3KO (black) mice, which were left untreated (control) or treated with LPS (1 μg/ml) or IL-4 (25 ng/ml). Twenty-four hours later, the RNA was extracted and analysed by quantitative RT-PCR. Inflammatory (**a**), anti-inflammatory mediators (**b**) and neurotrophic factors (**c**) were evaluated. *Gapdh* transcript was used as a house keeping for normalization. Data from three (*nrf2, bdnf*, *igf1*, *nt3*), five (*ym1*), six (*il1b*, *fizz1*, *gdnf*) and seven (*inos*, *arg1*) independent experiments is shown. Values are the mean ± SEM. *p < 0.05 by two-way ANOVA followed by Sidak’s post-hoc test

Since experiments commented above were carried out in WT or global DRD3KO mice, the effects observed could be due to the deficiency of DRD3 signalling in astrocytes or other kind of cells that normally express DRD3. To analyse the precise role of DRD3 signalling confined to glial cells in the production of mediators in response to inflammatory or anti-inflammatory environments, we next performed experiments in primary cultures of mixed glial cells containing both, astrocytes and microglia. For this purpose, primary cultures of mixed glial cells were generated from the midbrain/striatum of WT or DRD3-deficient newborn mice and challenged with pro-inflammatory or anti-inflammatory environments given by LPS or IL-4 respectively, and the extent of transcripts codifying for a panel of cytokines, enzymes and transcription factors related with the regulation of neuroinflammation was determined by quantitative RT-PCR (qRT-PCR). The results show that DRD3 deficiency in glial cells resulted in a selective increase in the transcription of the gene codifying for IL-1β and without effect in other mediators, enzymes or transcription factors evaluated in response to LPS (Fig. 8a–c). Conversely, the lack of DRD3 signalling in glial cells resulted in exacerbated transcription of the anti-inflammatory mediator Fizz1, without detectable effects in other mediators evaluated in response to an anti-inflammatory environment given by IL-4 (Fig. 8a–c).

**Fig. 9:**
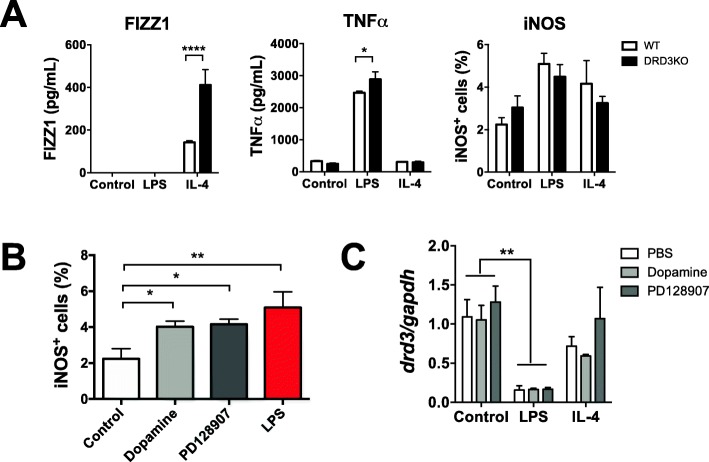
DRD3 signalling induces a pro-inflammatory profile in astrocytes. **a** Mixed glial cultures were generated from the midbrain/striatum structures obtained from WT (white) or DRD3KO (black) mice, which were left untreated (control) or treated with LPS (1 μg/ml) or IL-4 (25 ng/ml). Twenty-four hours later, the secretion of Fizz1 and TNF-α was determined in the supernatant of cultures by ELISA (left and middle panels) and the extent of iNOS expression (right panel) was determined by flow cytometry. Values are the mean ± SEM from triplicates. *p < 0.05; ****p < 0.00001 by two-way ANOVA followed by Sidak’s post-hoc test. **a** Astrocytes were generated from the midbrain/striatum structures obtained from WT mice. Cells were left untreated (control) or treated with dopamine (100 nM), a DRD3-selective agonist (PD128907, 20 nM) or LPS (1 μg/ml) for 24 h and the expression of iNOS was quantified in the GFAP^+^ population by flow cytometry. Numbers represent the frequency of cells iNOS^+^. Values are the mean ± SEM from triplicates. *p < 0.05; **p < 0.01 by one-way ANOVA followed by Tukey’s post-hoc test. **c** Astrocytes were generated from the midbrain/striatum structures obtained from WT mice. The cells were left untreated (control) or treated with LPS (1 μg/ml) or IL-4 (25 ng/ml) in the absence or presence of dopamine (100 nM) or PD128907 (20 nM) for 24 h. Afterwards, the RNA was extracted and the levels of *drd3* transcripts were evaluated by quantitative RT-PCR. *Gapdh* transcript was used as a house keeping for normalization. Values are the mean ± SEM from triplicates. **p < 0.01 by two-way ANOVA followed by Sidak’s post-hoc test. Representative data from one out of two independent experiments is shown

To confirm the most relevant differences obtained in mixed glial cultures at the level of protein, we next evaluated the production of IL-1β and Fizz1 in the supernatant of the cultures after 24 h of LPS or IL-4 treatment. The results show that DRD3 deficiency in glial cells leads to approximately three-fold higher production of Fizz1 in the supernatant in response to an anti-inflammatory environment given by IL-4 (Fig. 9a, left panel), thus confirming the results obtained at the levels of *fizz1* transcripts (Fig. 8b). Conversely, the concentration of IL-1β was undetectable under all conditions tested here (data not shown). To evaluate another relevant pro-inflammatory cytokine associated to neuroinflammation, we evaluated the production of TNFα in the supernatant of mixed glial cultures. Interestingly, the results show that DRD3 deficiency in glial cells leads to increased secretion of TNFα within the supernatant of the culture in response to a pro-inflammatory environment given by LPS treatment (Fig. 9a, middle panel). In addition, we determined the expression of the classic astrocytic pro-inflammatory marker iNOS in the GFAP^+^ population. In agreement with results obtained at the level of *inos* transcription (Fig. 8a), we did not observe differences in the extent of iNOS expression at the level of protein between astrocytes from both genotypes at any of the conditions tested (Fig. 9a, right panel). Thus, together, these results suggest that DRD3 signalling confined to astrocytes plays a dual role limiting the production of inflammatory cytokines in response to a pro-inflammatory environment, but also attenuating Fizz1 secretion by glial cells in response to anti-inflammatory cues.

To gain a further mechanistic insight of the role of DRD3 signalling in the pro-inflammatory response of astrocytes, we addressed the question of whether the direct DRD3 stimulation on astrocytes affects the pro-inflammatory profile of these cells and whether this signalling exerts regulation in DRD3 expression. For this purpose, we generated cultures of astrocytes isolated from the midbrain/striatum of WT mice and were treated with dopamine 100 nM (a concentration that selectively stimulates DRD3) or a DRD3-selective agonist (PD128097) for 24 h and the expression of the pro-inflammatory marker iNOS was evaluated by flow cytometry. As a positive control of inflammatory signal, astrocytes were treated with LPS. The results show that both dopamine and PD128907 induced a significant increase of iNOS expression, in a similar extent to that induced by LPS (Fig. 9b). Thus, these results indicate that DRD3 stimulation in astrocytes induces a pro-inflammatory profile. To determine whether DRD3 stimulation in astrocytes exerts regulation on DRD3 expression, next we treated astrocytes with dopamine or PD128907 in the absence of an additional challenge or in response to a pro-inflammatory environment given by LPS treatment or in response to an anti-inflammatory environment given by IL-4 treatment, and the levels of *drd3* transcription were evaluated by qRT-PCR. The results show that DRD3 stimulation by dopamine or PD128907 did not exert a significant regulation on *drd3* transcription under any of the conditions tested (Fig. 9c). However, the *drd3* transcription was significantly reduced in response to a pro-inflammatory environment given by LPS treatment (Fig. 9c). *Drd3* transcription was not affected in response to an anti-inflammatory environment given by IL-4 treatment (Fig. 9c). Taken together, these results indicate that DRD3 stimulation induces pro-inflammatory signals in astrocytes similar to those exerted by LPS; however, only a classic pro-inflammatory challenge such as that induced by LPS, but not that induced by DRD3 signalling, exerts downregulation of *drd3* transcription.

## Discussion

According to our previous results showing a selective *drd3* transcription in astrocytes but not in microglia [31], the present study shows the expression of DRD3 at the protein level in primary astrocytes and not in microglial cells (Fig. 1). Interestingly, the DRD3 immunoreactivity in astrocytes is associated with a clustered pattern, resembling the expression pattern observed for those proteins contained in lipid-rafts, which facilitates the cooperation and interaction between different cell-surface proteins to trigger intracellular signalling [43]. Nevertheless, since the protocol used for immunofluorescence analyses involves the permeabilization of cells, we cannot rule out the possibility that clustered pattern of DRD3 expression is due to the localization into intracellular stores.

A recent study analysed the dynamic of acquisition of pro-inflammatory and anti-inflammatory phenotypes by glial cells upon neuroinflammation induced by the systemic administration of LPS [39]. Interestingly, the authors found that microglial cells acquire the M1 pro-inflammatory phenotype at early time-points after LPS-induced neuroinflammation. At late time-points, the M1 phenotype was dramatically turned off and, conversely, the M2 anti-inflammatory phenotype was upregulated in microglial cells. Of note, the acquisition of the M2 phenotype at late time-points was accompanied by a sustained activation of astrocytes [39]. According to that study, in the present work, we observed a quick increase of the M1-to-M2 ratio in the phenotype of microglia at time-points as early as 4 h in LPS-treated mice (Additional file 1: Figure S5), which was followed later by the activation of astrocytes (Figs. 5 and 6). We did not observe the reduction of the M1 phenotype and the reciprocal increase in the acquisition of M2 phenotype in microglial cells accompanied by the sustained activation of astrocytes probably because we did not analyse glial phenotypes at time-points late enough to appreciate this process. Interestingly, our data here shows that, despite the increased expression of the activation marker Iba1 was abolished in DRD3KO mice at early time-points after LPS treatment (Fig. 3), DRD3 deficiency resulted in a faster increase of the M1-to-M2 ratio in the phenotype of microglial cells (Additional file 1: Figure S5) and unresponsiveness of astrocytes upon neuroinflammation induced by LPS (Figs. 5, 6 and Additional file 1: Figure S5). Thus, our results suggest that DRD3 signalling plays a critical role in the activation of microglia and astrocytes and in the regulation of the functional phenotype of microglial cells in the context of neuroinflammation.

DRD3 has been previously described to be expressed in astrocytes of mouse [30, 31], rat [29] and human [44] origin. Of note, astrocytic expression of DRD3 was significantly exacerbated in Alzheimer’s disease patients, a neurodegenerative disorder involving chronic neuroinflammation [44]. According to the results obtained here and to our previous study [31], this increased expression of DRD3 in astrocytes could represent a pathological mechanism exacerbating neuroinflammation and perpetuating the chronic progression of this disorder. Importantly, DRD3 has been found to be coupled to the inhibition of cAMP production [26]. In this regard, cAMP has been found to be an important intracellular mediator favouring the transition from pro-inflammatory phenotypes to anti-inflammatory phenotypes in glial cells [45]. According to this idea, the purinergic receptor P2Y_12_, another G-protein coupled receptor (GPCR) coupled to the inhibition of cAMP and expressed in glial cells, has been shown to favour glial activation and chemotaxis to the injured site upon neuron damage [46].

In apparent controversy, previous studies have indicated that dopaminergic signalling in astrocytes attenuates neuroinflammation, and consequently exerts a protective effect in neurodegeneration [19, 20]. In this regard, it has been shown that mice harbouring DRD2-deficient astrocytes develop an exacerbated inflammatory response and display increased loss of dopaminergic neurons in the substantia nigra upon treatment with MPTP. Conversely, the treatment of WT mice with a DRD2 agonist exerted a protective effect reducing the extent of neuroinflammation and neurodegeneration in MPTP-treated mice. Interestingly, this anti-inflammatory effect triggered by DRD2 stimulation was mediated by the increased expression of αB-crystallin in astrocytes, which regulates innate immunity [20]. On the other hand, it has been shown that DRD1 signalling in astrocytes and microglial cells regulates negatively the activation of the inflammasome NLRP3, thus attenuating the production of inflammatory cytokines and neurodegeneration induced by MPTP in mice. Accordingly, the administration of a DRD1 agonist in MPTP mice significantly reduced the loss of nigral dopaminergic neurons. Interestingly, this anti-inflammatory effect exerted by DRD1 signalling was mediated by an increase on cAMP levels, which induces the ubiquitination and subsequent degradation of the NLRP3 inflammasome [19]. It is noteworthy that these anti-inflammatory effects exerted by dopaminergic signalling in astrocytes are mediated by low-affinity DRs, which display Ki values in the micromolar order (≈1705 nM for DRD2 and ≈2340 nM for DRD1) [47, 48]. Conversely, our results here indicate that dopaminergic signalling triggered by the stimulation of DRD3, which display the highest affinity for dopamine (Ki ≈ 27 nM) [49], promotes a pro-inflammatory behaviour in astrocytes. Thereby, taken together, our findings and previous reports addressing the role of dopaminergic signalling in astrocytes in the context of inflammation, the evidence suggests that high dopamine levels (in the order of μM) would exert anti-inflammatory effects in astrocytes, whereas low dopamine levels (in the order of nM) would selectively stimulate DRD3 favouring neuroinflammation.

Previous studies have shown a fundamental role of DRD3 signalling in neurodegeneration associated to Parkinson’s disease. In this regard, two independent laboratories demonstrated that genetic deficiency of DRD3 resulted in a strong protection from the degeneration of the dopaminergic neurons of the *substantia nigra* in mice upon acute intoxication with MPTP [33, 34]. Moreover, in recent studies, we show how the systemic administration of a DRD3 selective antagonist attenuated the neurodegeneration of the nigrostriatal pathway in two different mouse models of Parkinson’s disease, including the chronic intoxication with MPTP and probenecid and the stereotaxic administration of 6-hydroxydopamine [25, 31]. According to these studies, the present results show that DRD3 deficiency dampens neuroinflammation, one of the main processes involved in the triggering neurodegeneration associated to Parkinson’s disease [41]. Since previous evidence addressing the role of DRD3 signalling in neurodegeneration have shown that general genetic deficiency or systemic antagonism of DRD3 results in the inhibition of neuroinflammation and neurodegeneration associated to animal models of Parkinson’s disease, DRD3 signalling from different sources could be relevant in this effect. In this regard, DRD3 expressed in CD4^+^ T cells has been described to play a relevant role favouring the development of Parkinson’s disease in mice intoxicated with MPTP [33]. Furthermore, DRD3 has been found to be expressed in several structures of the brain, including the *substantia nigra*, ventral tegmental area, cerebellum, nucleus accumbens and olfactory tubercle [50–53]. Thus, DRD3 expressed in these areas of the brain could be contributing to promote neuroinflammation in different conditions. Since our results here show that DRD3 is selectively expressed in astrocytes but not in microglia (Fig. 1), and its deficiency resulted in a significant alteration in the pattern of inflammatory and anti-inflammatory mediators produced in cultures of mixed glial cells (Figs. 8 and 9), an important contribution of DRD3-mediated effect in neuroinflammation in vivo can be attributed to DRD3 expressed in astrocytes. However, DRD3 has also been found to be expressed in pre- and post-synaptic neurons of dopaminergic circuits where this receptor not only mediates dopaminergic neurotransmission but also regulates synthesis and release of dopamine [35]. Thereby, a potential contribution of neuronal DRD3 to the pro-inflammatory effect mediated by DRD3 in neuroinflammation in vivo cannot be ruled out.

The results obtained in this study raise the question of how DRD3 signalling in astrocytes is involved in microglia-astrocyte cross-talk. In this regard, it is important to consider the mechanisms mediated by glutamate and TNF-α, two major signals of microglia-mediated neurotoxicity [4]. When Toll-like receptors (TLRs) are stimulated in microglial cells, such as the case of LPS-mediated stimulation of TLR4, a pro-inflammatory program is triggered which includes the production of TNF-α, and the increased expression of glutaminase that, in turn, results in enhanced secretion of glutamate though the cystine/glutamate antiporter system [54, 55]. High levels of TNF-α and glutamate in turn might induce neuronal death through the stimulation of TNFR1 and N-methyl d-aspartate (NMDA) receptors in neurons [56, 57]. Moreover, TNF-α induces increased glutaminase expression in microglial cells, thereby enhancing glutamate production, whilst glutamate stimulates AMPA (α-amino-3-hydroxy-5-methyl-4-isoxazolepropionic acid) receptors in glial cells, exacerbating TNF-α production [58, 59]. Thus TNF-α and glutamate act as two synergistic inflammatory mediators produced by microglia. Of note, it has been shown that TNF-α might act on astrocytes inducing further production of TNF-α and other inflammatory molecules such as IL-6 and monocyte chemoattractant protein 1 (MCP-1) [60]. Furthermore, TNF-α inhibits the uptake of glutamate by astrocytes, which constitute the main mediators of glutamate clearance in homeostatic conditions [54, 58]. In this way, astrocytes represent important players in neuroinflammation, which provide microglia with a positive feedback exacerbating glutamate- and TNF-α-mediated neurotoxicity. Importantly, it has been described that stimuli that induce enhanced levels of cAMP in astrocytes attenuate TNF-α-mediated inflammatory effects in these cells [60]. Thereby, DRD3 signalling, which involves a reduction of cAMP levels [26], would favour the pro-inflammatory behaviour of astrocytes, potentiating the detrimental effect exerted by microglial cells in neuroinflammation and neurodegeneration.

Our findings here show that one of the main mediators affected by DRD3 deficiency in glial cells was Fizz1, which has been associated with M2 microglia [45, 61]. According to the strong exacerbation in the production of Fizz1 induced in the brain by the intraventricular injection of IL-4 [61], our in vitro analyses show that the stimulation of glial cells with IL-4 triggers a potent production of Fizz1 (Fig. 8b). Moreover, the genetic deficiency of DRD3 in glial cells resulted in a striking exacerbation in the production of Fizz1 in response to IL-4 (Figs. 8b and 9a). In this regard, a potent anti-inflammatory effect has been described for IL-4 combined with agents that promotes cAMP production dampening neuroinflammation [45]. Accordingly, the deficiency of DRD3, which is normally coupled to G_i/o_-alpha subunit, should result basally in increased cAMP production that could explain the exacerbated IL-4-mediated increase in Fizz1. Interestingly, in addition to in vitro analyses carried out in isolated glial cells, our in vivo experiments showed a marked increase in the basal production of Fizz1 in the midbrain/striatum of DRD3-deficient mice (Fig. 7b), thus suggesting that DRD3 deficiency would give a higher anti-inflammatory environment to the brain parenchyma, even in the absence of an inflammatory stimulus. Interestingly, Fizz1 has been shown to exert a potent anti-inflammatory effect in T-helper 2 (Th2)-mediated lung inflammation [62]. On the other hand, we have recently found that DRD3 deficiency results in exacerbated Th2-mediated airways allergy in response to house-dust mite derived antigens [21]. Thus, it seems that lack of DRD3 triggers not only the mechanisms to exert stronger Th2-mediated inflammation but also those mechanisms involved in the attenuation of Th2-mediated responses.

**Fig. 10:**
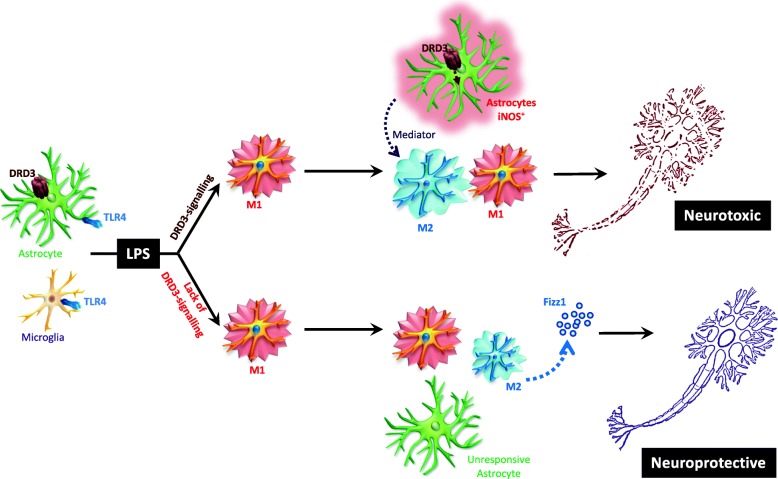
Proposed model. Upon systemic LPS-challenge, TLR4 is stimulated in microglial cells favouring microglial activation and the acquisition of M1 phenotype. Top arm: Subsequently, TLR4 stimulation and the co-stimulation of DRD3 in astrocytes promotes their activation and the acquisition of a pro-inflammatory profile, characterized by high iNOS expression. Furthermore, DRD3 signalling in inflammatory astrocytes induces the generation of a mediator (still non-identified) which attenuates Fizz production by M2 microglia, thus favouring the function of M1 microglia and their neurotoxic consequences. Bottom arm: TLR4 stimulation in the absence of DRD3 signalling in astrocytes seems to render these cells unresponsive. Thereby, the absence of DRD3 signalling results in increased production of Fizz1 and consequently in a neuroprotective environment

It is noteworthy that Fizz1 was barely detectable in cultures of astrocytes exposed to an anti-inflammatory environment given by IL-4 treatment (data not shown), suggesting that Fizz1 detected in mixed glial cultures was produced by microglial cells. Considering our previous [31] and present results indicating that DRD3 is expressed selectively in astrocytes but not in microglial cells (Fig. 1), the present study suggests that DRD3 stimulation in astrocytes induces the expression, synthesis and/or release of a mediator that dampens the production of Fizz1 by microglial cells (Fig. 10). Despite we did not detect this astrocyte-derived mediator in our study, it is tempting to speculate that such a mediator should be an inhibitor of NF-κB [63, 64], an inducer of STAT3 or STAT6 phosphorylation [64, 65] or an activator of the RORα/AMPKα-axis [66] in microglial cells, all of them signalling pathways that results in increased production of Fizz1 by microglia. Despite most likely one or more of these signalling pathways are induced by the putative astrocyte-derived mediator, further experimental work is necessary to identify such an astrocyte-derived mediator.

It is noteworthy that transcriptional levels of *Fizz1* were strongly increased in the brain of DRD3-deficient mice in comparison with those found in the brain of WT mice upon basal conditions (Fig. 7b). Conversely, *Fizz1* transcription in glial cultures was similar among both genotypes in basal conditions (Fig. 8b). Thereby, it is likely that DRD3 signalling in another cell type, different of astrocytes and microglia, would be attenuating Fizz1 production. In this regard, DRD3 signalling in neurons could be affecting the cross-talk between neurons and microglia. For instance, in healthy conditions, neurons produce several soluble mediators and cell-surface proteins that exert anti-inflammatory effects on microglial cells, including the production of fractalkine, CD22, CD200 and SIRP-α [4]. Thus, DRD3 signalling in neurons could exert a negative regulation of one or more of these anti-inflammatory signals given to microglial cells and consequently reducing Fizz1 production by those cells. Nevertheless, this hypothesis should be experimentally evaluated.

## Conclusions

Our findings here show genetic and pharmacologic evidence demonstrating how dopaminergic signalling mediated through DRD3 regulates the dynamic of the acquisition of pro-inflammatory and anti-inflammatory features by astrocytes and microglia, ultimately promoting neuroinflammation. Lack of DRD3 signalling induced unresponsiveness of astrocytes with attenuated microglial activation and an early increase of the M1-to-M2 ratio in the phenotype of microglia in response to inflammatory stimuli, which finally resulted in dampening neuroinflammation. The mechanistic analysis showed that DRD3 deficiency resulted in exacerbated expression of the anti-inflammatory mediator Fizz1 in glial cells both in vitro and in vivo (see the proposed model in Fig. 10). This is the first study showing how DRD3 signalling regulates the dynamics of glial activation, impacting in the final outcome of neuroinflammation.

## Supplementary information

**Additional file 1: Figure S1.** Analysis of astrocyte activation in different areas of the mouse brain upon LPS treatment. (A) Wild-type mice received an i.p. injection of PBS (left panel) or 5 mg/kg of LPS (right panel) and 3h later were sacrificed and immunofluorescence analysis was performed in brain slices. Specific DRD3 (green) and GFAP (red) immunostaining was performed as indicated in the legend of Fig. 2. Nuclei were stained with DAPI (blue). Representative images of brain sections showing DRD3-immunostaing (top panels) and GFAP-immunostaining (middle panels). Pseudocolored image of a brain section showing the intensity of GFAP-associated immunoreactivity (bottom panel). Different regions of interest (ROI) were selected in different brain areas from LPS-treated mice (shown in white-framed squares; bottom-right panel). (**B**) High resolution images of ROIs selected (A; bottom-right panel) showing GFAP-immunostaining in different brain areas including cortex (ROI 1 and 2), subhypotalamic zone (ROI 3), corpus callosum (ROI 4), cerebral nuclei striatum (ROI 5) and cerebral nuclei pallidum (ROI 6). **Figure S2.** Systemic inflammation induced by LPS triggers the increase of inflammatory cytokines in the brain. WT mice were treated with an i.p. injection of LPS (5 mg/kg) or PBS (Control). 4h or 24h later, the midbrain/striatum structures were isolated, disaggregated, and the RNA was extracted and analysed by quantitative RT-PCR. (**A**) The transcript for TNF-α was determined 24 h after LPS administration. ***, p<0.001 by two-tailed unpaired Student’s *t*-test. (**B**) The transcript for IL-1β was quantified after 4 and 24 h after LPS administration.**, *p*<0.01; ***, *p*<0.0001 by one-way ANOVA followed by Tukey’s *post-hoc* test. (A and B) *Gapdh* transcript was used as a house keeping for normalization. Data from 4-8 mice per group is shown. Values are the mean ± SEM. **Figure S3.** Genetic deficiency or pharmacologic antagonism of DRD3-signalling reduces the M1-to-M2 ratio of microglial cells in the midbrain of mice undergoing systemic inflammation induced by LPS. Associated to Fig. 4. Wild-type (WT) or DRD3 knockout (DRD3KO) mice were pre-treated or not with an i.p. injection of a DRD3-selective antagonist (PG01037; 30 mg/kg) and 1h later received an i.p. injection of LPS (5 mg/kg) or PBS. 24h after LPS administration, the midbrain/striatum structures were isolated, disaggregated, and M1 (CD16/32^+^CD206^-^ cells) and M2 (CD16/32^+^CD206^+^ cells) phenotypes were analysed in living (ZAq^-^) microglial cells (CD11b^+^ CD45^+^) by flow cytometry. Representative contour-plots are shown. Numbers in red and blue indicate the percentage of M1 (CD16/32^+^CD206^-^ cells) and M2 (CD16/32^+^CD206^+^ cells) microglia in each sample. **Figure S4.** Similar density of expression of microglial markers in the midbrain of mice WT and DRD3-deficient mice undergoing systemic inflammation induced by LPS. Wild-type (WT) or DRD3 knockout (DRD3KO) mice were pre-treated or not with an i.p. injection of a DRD3-selective antagonist (PG01037; 30 mg/kg) and 1h later received an i.p. injection of LPS (5 mg/kg) or PBS. 24h after LPS administration, the midbrain/striatum structures were isolated, disaggregated, and different molecular markers were analysed in microglial cells by flow cytometry. The density of CD16/32, CD206, CD11b and CD45 was determined as the mean-fluorescence intensity (MFI) in the population of living (ZombieAqua^-^) microglial cells (CD11b^+^ CD45^+^). Top panels show data from 4-5 mice per group. Each symbol represents a WT (white) or a DRD3KO (black) animal. In each experimental group, the line and error bars represent the mean ± SEM respectively. *, *p*<0.05; **, *p*<0.01; ***, *p*<0.001; ****, *p*<0.0001 by one-way ANOVA followed by Tukey’s *post-hoc* test. Bottom panels show representative histograms. **Figure S5.** Similar behaviour of microglia and astrocytes in the midbrain of WT and DRD3KO mice at early time-points after the induction of systemic inflammation triggered by LPS. WT or DRD3KO mice were treated with an i.p. injection of LPS (5 mg/kg) or PBS (Control). 4h later, the midbrain/striatum structures were isolated, disaggregated, and the inflammatory and anti-inflammatory phenotypes of microglia (**A**) and astrocytes (**B**) were analysed by flow cytometry as described in Figs. 4 and 6 respectively. (A and B) Top panels show representative contour-plots indicating the percentage of pro-inflammatory glia (red numbers) and anti-inflammatory glia (blue numbers). Bottom panels show the quantification of the frequencies of inflammatory (left-bottom panels) and anti-inflammatory (middle-bottom panels) phenotypes and the inflammatory-to-anti-inflammatory ratio (right-bottom panels). Data from 4 mice per group is shown. Each symbol represents a WT (white) or a DRD3KO (black) animal. In each experimental group, the line and error bars represent the mean ± SEM, respectively. *, *p*<0.05; **, *p*<0.01; ***, *p*<0.001 by one-way ANOVA followed by Tukey’s *post-hoc* test. **Figure S6.** Similar density of expression of astrocytic markers in the midbrain of wild-type and DRD3-deficient mice undergoing systemic inflammation induced by LPS. WT or DRD3KO mice were treated with an i.p. injection of LPS (5 mg/kg) or PBS (Control). 24h later, the midbrain/striatum structures were isolated, disaggregated, and astrocytic markers were analysed by flow cytometry. The density of iNOS (left panels), Arg1 (middle panels) and GFAP (right panels) was determined as the mean fluorescence intensity (MFI) in the population of living (ZombieAqua^-^) astrocytes (GFAP^+^ cells). Top panels show the quantification from 8 mice per group. Each symbol represents a WT (white) or a DRD3KO (black) animal. In each experimental group, the line and error bars represent the mean ± SEM respectively. No significant differences were found among the different experimental groups. Bottom panels show representative histograms.

## Data Availability

The datasets used and/or analysed during the current study are available from the corresponding author on reasonable request.

## Abbreviations

BDNF: Brain-derived neurotrophic factor
DAT: Dopamine transporter
D*n*R: Dopamine receptor *n*
D*n*RKO: D*n*R knockout
DRs: Dopamine receptors
FBS: Foetal bovine serum
GAPDH: Glyceraldehyde 3-phosphate dehydrogenase
GDNF: Glial cell-derived neurotrophic factor
GFAP: Glial fibrillary acidic protein
GPCR: G-protein coupled receptors
HBSS: Hank’s balanced salt solution
IGF-1: Insulin-like growth factor 1
iNOS: Inducible nitric oxide synthase
MPTP: 1-Methyl-4-phenyl-1,2,3,6-tetrahydropyridine
RNS: Reactive nitrogen species
ROS: Reactive oxygen species
SN*pc*: *Substantia nigra pars compacta*
TH: Tyrosine hydroxylase
TLR*n*: Toll-like receptor *n*
TLRs: Toll-like receptors
