# Supplementary material for: Dopamine receptor D3 signalling in astrocytes promotes neuroinflammation
Source: J Neuroinflammation. 2019 Dec 6;16:258. doi: 10.1186/s12974-019-1652-8 (PMC6896356; doi:10.1186/s12974-019-1652-8)

SUPPLEMENTARY FIGURES

Dopamine receptor D3 signalling in astrocytes promotes neuroinflammation

Andro Montoya, Daniela Elgueta, Javier Campos, Ornella Chovar, Paulina Falcón, Soledad Matus, Iván Alfaro, María Rosa Bono, Rodrigo Pacheco

Figure S1. Montoya et al

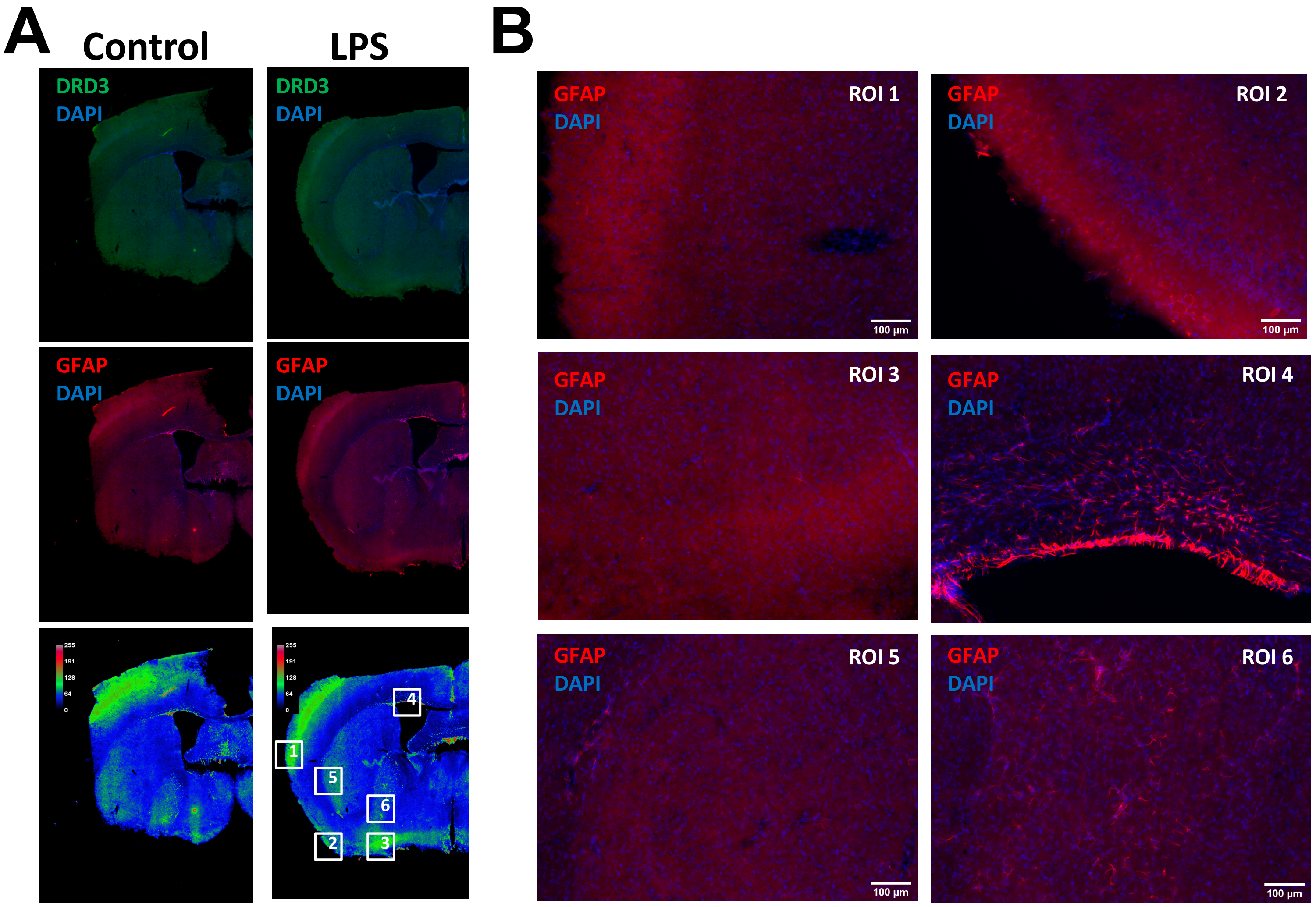

Figure S2. Montoya et al

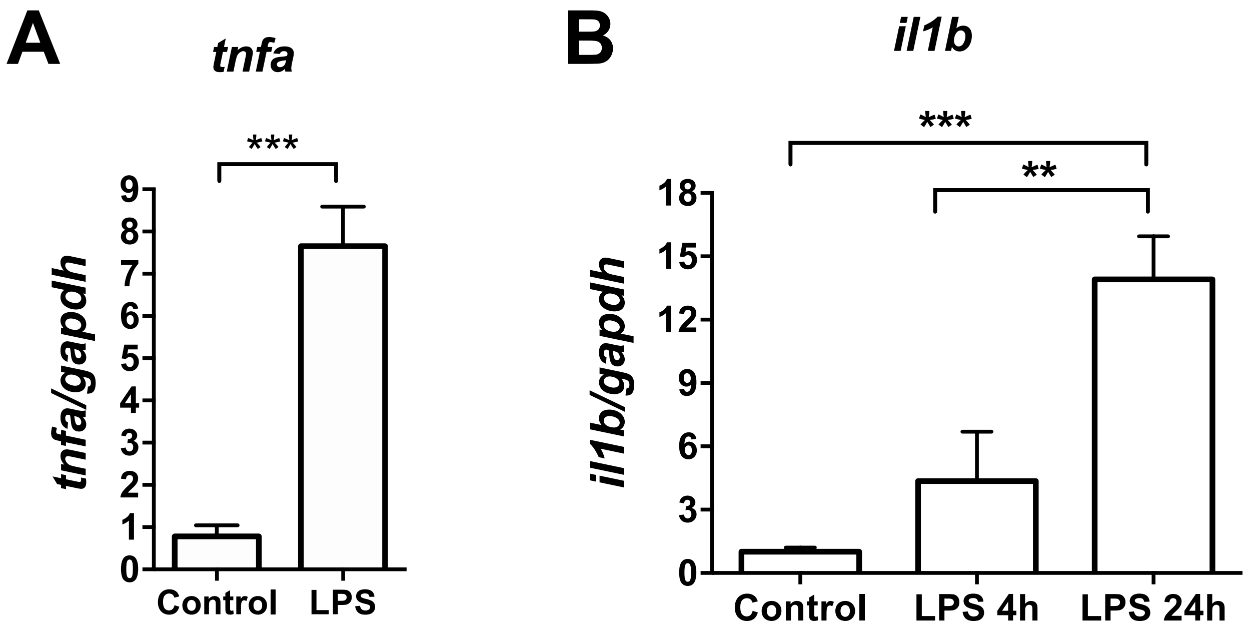

Figure S3. Montoya et al

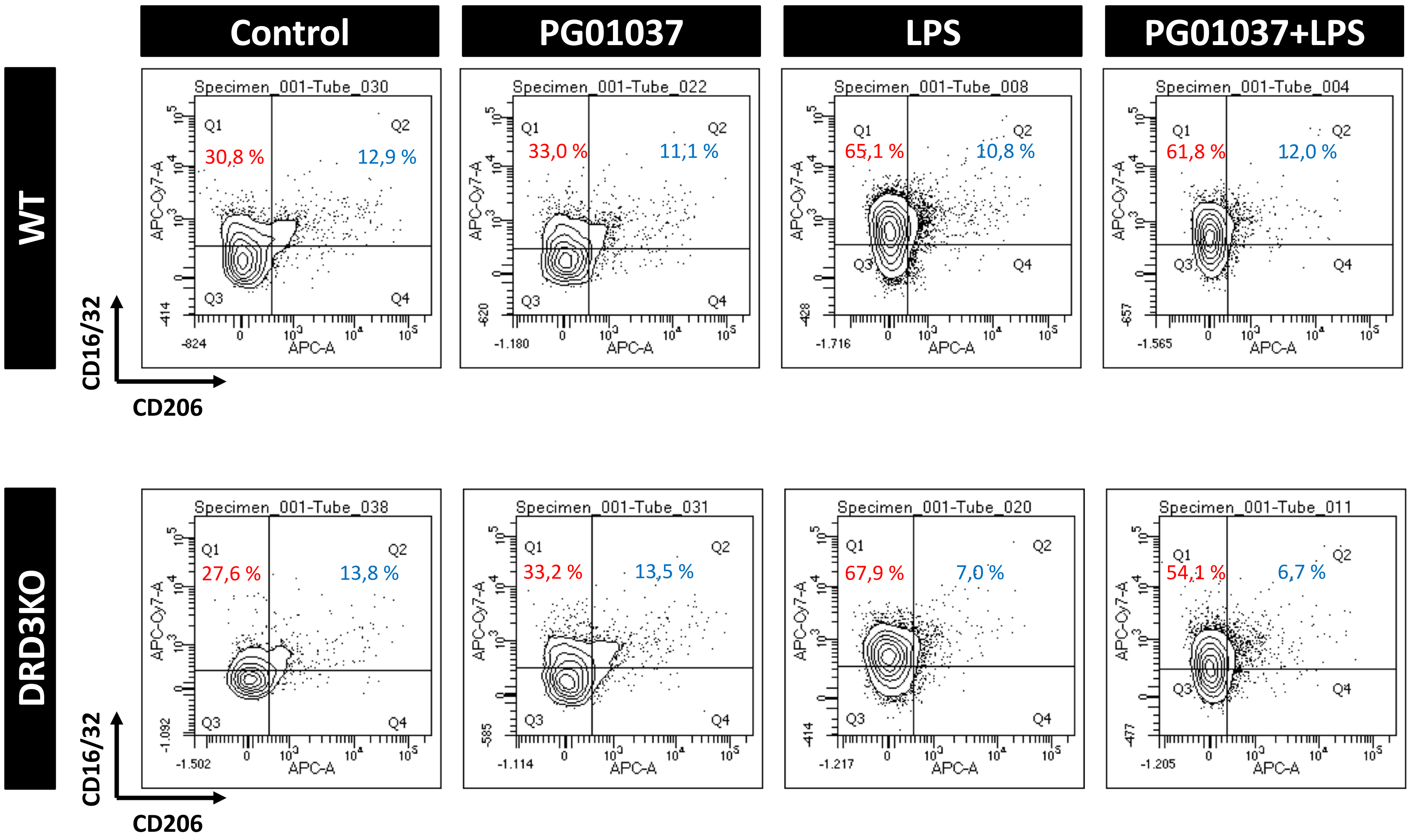

Figure S4. Montoya et al

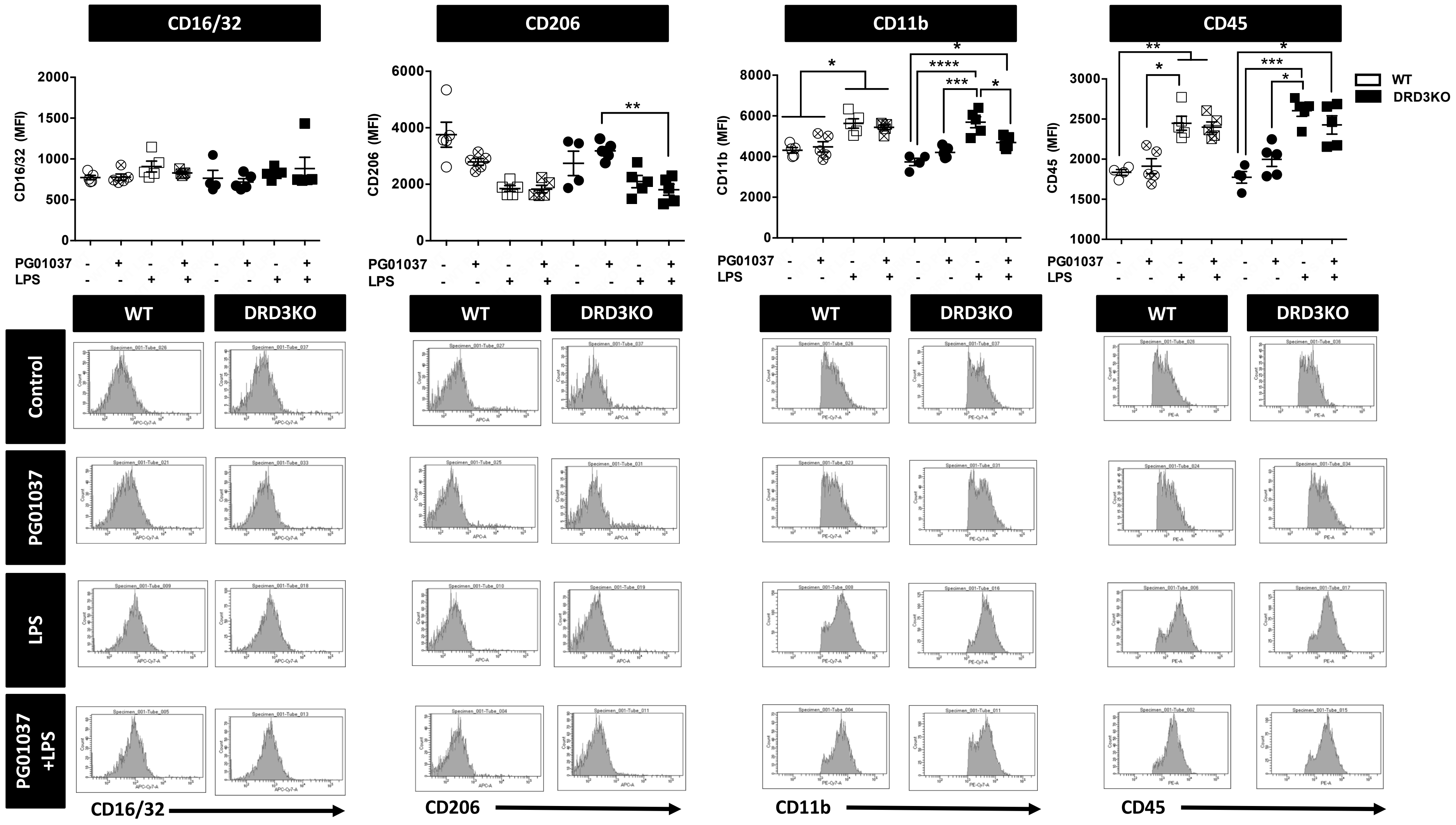

Figure S5. Montoya et al

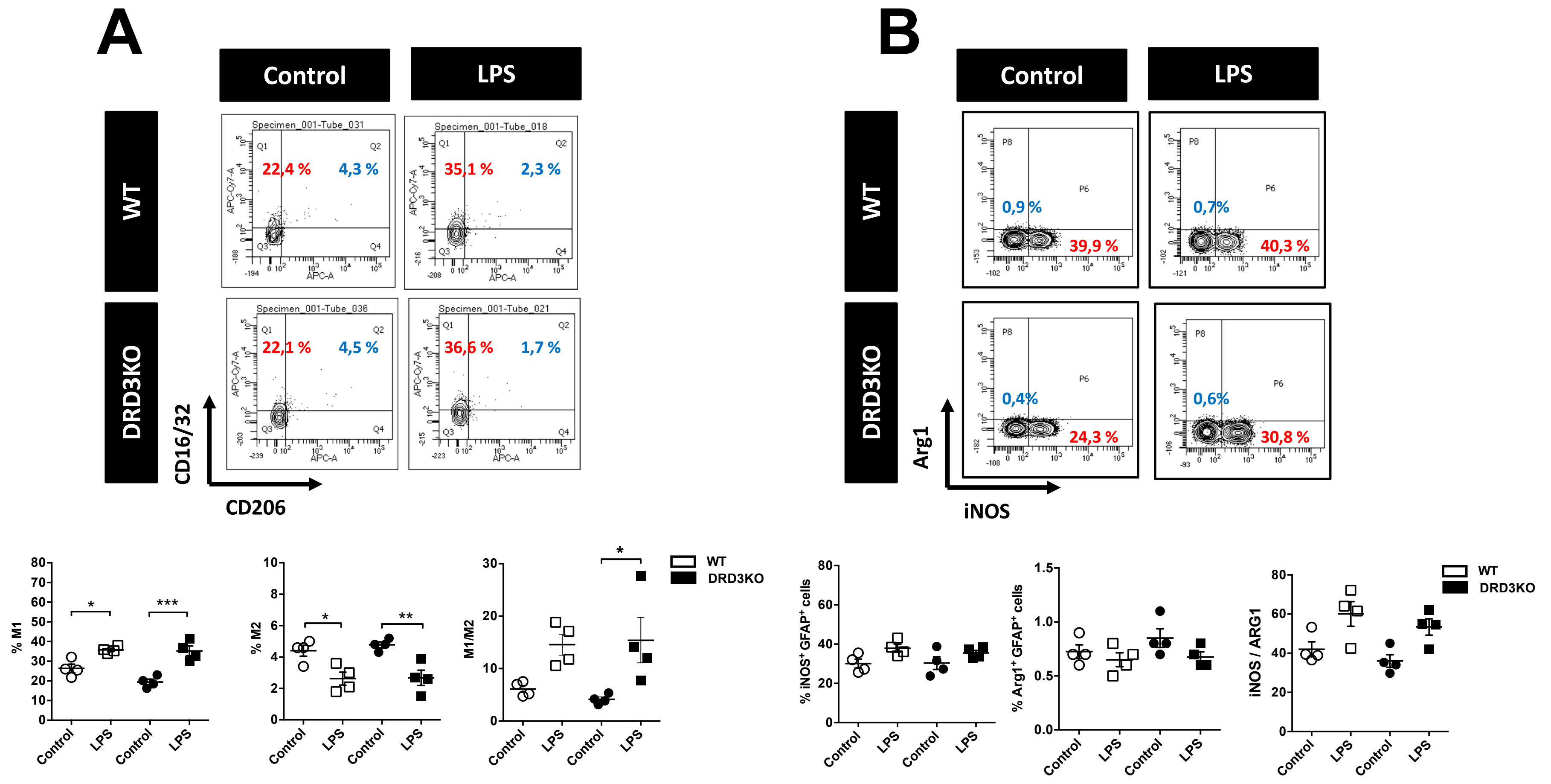

Figure S6. Montoya et al

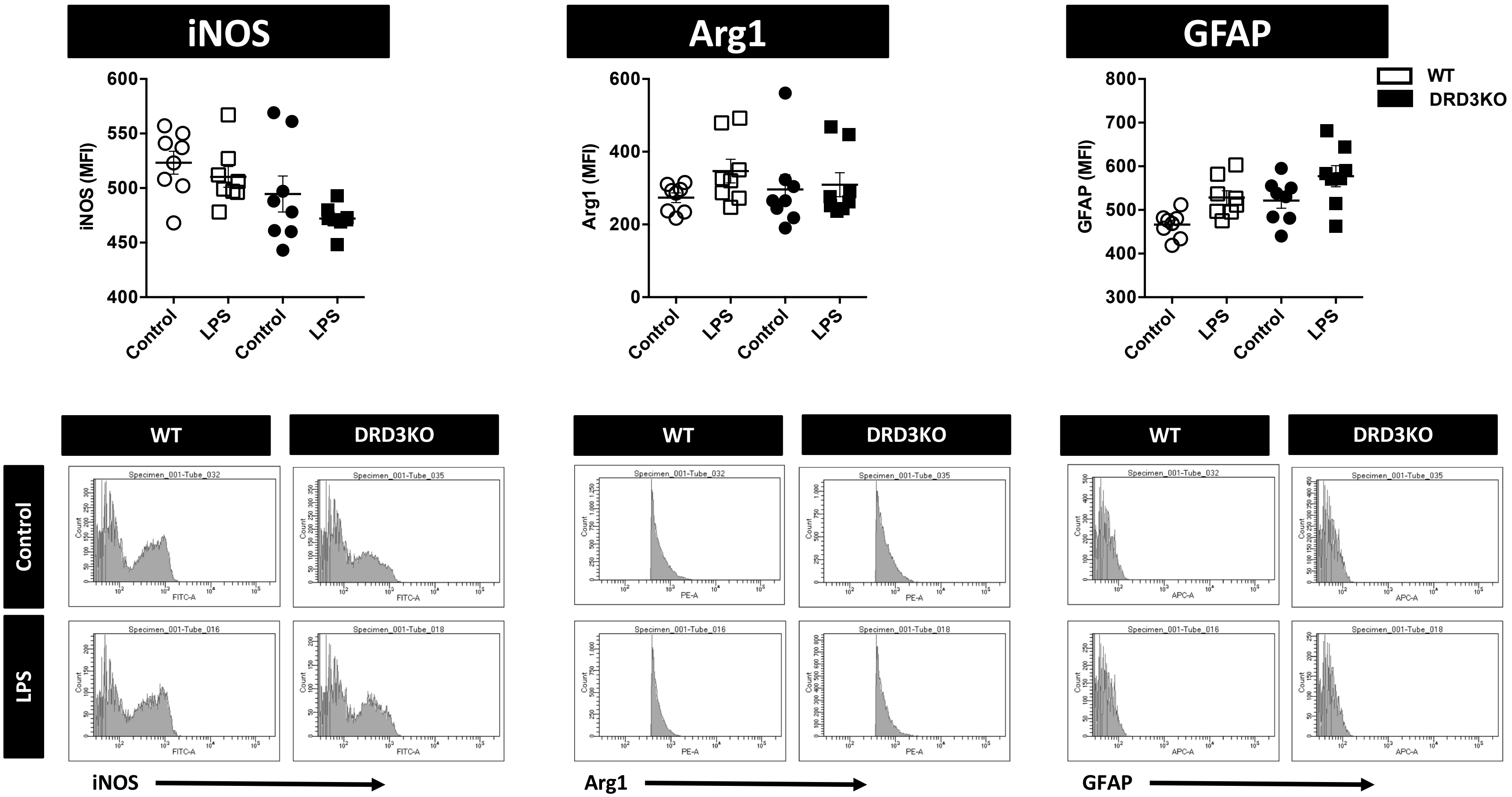

Supplement: Supplementary file 1 — Additional file 1: Figure S1. Analysis of astrocyte activation in different areas of the mouse brain upon LPS treatment. (A) Wild-type mice received an i.p. injection of PBS (left panel) or 5 mg/kg of LPS (right panel) and 3h later were sacrificed and immunofluorescence analysis was performed in brain slices. Specific DRD3 (green) and GFAP (red) immunostaining was performed as indicated in the legend of Fig. 2. Nuclei were stained with DAPI (blue). Representative images of brain sections showing DRD3-immunostaing (top panels) and GFAP-immunostaining (middle panels). Pseudocolored image of a brain section showing the intensity of GFAP-associated immunoreactivity (bottom panel). Different regions of interest (ROI) were selected in different brain areas from LPS-treated mice (shown in white-framed squares; bottom-right panel). (B) High resolution images of ROIs selected (A; bottom-right panel) showing GFAP-immunostaining in different brain areas including cortex (ROI 1 and 2), subhypotalamic zone (ROI 3), corpus callosum (ROI 4), cerebral nuclei striatum (ROI 5) and cerebral nuclei pallidum (ROI 6). Figure S2. Systemic inflammation induced by LPS triggers the increase of inflammatory cytokines in the brain. WT mice were treated with an i.p. injection of LPS (5 mg/kg) or PBS (Control). 4h or 24h later, the midbrain/striatum structures were isolated, disaggregated, and the RNA was extracted and analysed by quantitative RT-PCR. (A) The transcript for TNF-α was determined 24 h after LPS administration. ***, p<0.001 by two-tailed unpaired Student’s t-test. (B) The transcript for IL-1β was quantified after 4 and 24 h after LPS administration.**, p<0.01; ***, p<0.0001 by one-way ANOVA followed by Tukey’s post-hoc test. (A and B) Gapdh transcript was used as a house keeping for normalization. Data from 4-8 mice per group is shown. Values are the mean ± SEM. Figure S3. Genetic deficiency or pharmacologic antagonism of DRD3-signalling reduces the M1-to-M2 ratio of microglial cel [file 12974_2019_1652_MOESM1_ESM.pdf]
